# Accelerometry-Based Digital Gait Characteristics for Classification of Parkinson's Disease: What Counts?

**DOI:** 10.1109/OJEMB.2020.2966295

**Published:** 2020-02-14

**Authors:** Rana Zia Ur Rehman, Christopher Buckley, Maria Encarna Micó-Amigo, Cameron Kirk, Michael Dunne-Willows, Claudia Mazzà, Jian Qing Shi, Lisa Alcock, Lynn Rochester, Silvia Del Din

**Affiliations:** ^1^ Translational and Clinical Research InstituteNewcastle University 204292 Newcastle Upon Tyne NE4 5PL U.K.; ^2^ School of Mathematics, Statistics, and PhysicsNewcastle University 98458 Newcastle Upon Tyne NE1 7RU U.K.; ^3^ Department of Mechanical Engineering and INSIGNEO Institute for in silico MedicineUniversity of Sheffield 7315 Sheffield S10 2TN U.K.; ^4^ Newcastle upon Tyne Hospitals NHS Foundation Trust 5983 Newcastle Upon Tyne NE7 7DN U.K.

**Keywords:** Classification, Machine Learning, Digital Gait, Parkinson's disease, Partial least square-discriminant analysis (PLS-DA)

## Abstract

*Objective:* Gait may be a useful biomarker that can be objectively measured with wearable technology to classify Parkinson's disease (PD). This study aims to: (i) comprehensively quantify a battery of commonly utilized gait digital characteristics (spatiotemporal and signal-based), and (ii) identify the best discriminative characteristics for the optimal classification of PD. *Methods:* Six partial least square discriminant analysis (PLS-DA) models were trained on subsets of 210 characteristics measured in 142 subjects (81 people with PD, 61 controls (CL)). *Results:* Models accuracy ranged between 70.42-88.73% (AUC: 78.4-94.5%) with a sensitivity of 72.84-90.12% and a specificity of 60.3-86.89%. Signal-based digital gait characteristics independently gave 87.32% accuracy. The most influential characteristics in the classification models were related to root mean square values, power spectral density, step velocity and length, gait regularity and age. *Conclusions:* This study highlights the importance of signal-based gait characteristics in the development of tools to help classify PD in the early stages of the disease.

## I. Introduction

Parkinson's disease (PD) is the second most common neurodegenerative disease after Alzheimer's disease [1]. PD presents a combination of motor and non-motor symptoms that collectively can cause functional disability, loss of independence and reduced quality of life [2]. The heterogeneity of PD creates significant problems for accurate diagnosis, particularly in the early disease stages where symptoms may be very subtle [3]. Diagnostic accuracy to differentiate PD from other neurological disorders by movement disorder specialists ranges between 74% and 80% [4]. PD state markers (status i.e., with or without PD) with strong sensitivity and specificity also have potential to act as trait markers (detection of disease in its prodromal stage). They are therefore of paramount importance because they could contribute towards timely and accurate diagnosis and clinical management [5].

Gait is a potential state and trait marker because gait impairments present in very early disease [6], precede the onset of overt motor signs and evolve more rapidly than other motor features of PD [7]. Tools to objectively quantify discrete gait characteristics include pressure insoles/mats, 3D motion capture, force plates, electromyography, and instrumented walkways/treadmills [8]. Although they are essential to accurately characterize gait impairments in clinical populations, their routine use is limited to research settings due to cost and the expertise required to use them [9], [10]. If gait assessments are to provide state, and potentially trait markers for PD, development of tools that are highly specific and sensitive to PD, whilst remaining clinically and ecologically viable, are essential.

Wearable devices such as accelerometers provide a solution to this challenge. They are capable of quantifying digital gait characteristics objectively in clinical/laboratory-based settings as well as in real-world conditions [11]. Accelerometers can capture spatiotemporal characteristics similar to other gait analysis tools [12]. They can also capture gait continuously over long distances/durations, which drastically increases the opportunity to extract additional meaningful information. For example, using signal processing techniques, time and frequency domain analysis can quantify signal magnitude, regularity, complexity, smoothness, and symmetry [13]–[15]. These alternative signal derived gait characteristics may provide complementary/superior state markers in early PD [16] and in objective monitoring of PD [17]. The optimal characteristic or combination therefore remains unclear.

Tools are needed to evaluate the optimal combination of characteristics for use in PD in order to improve disease classification. Data driven modelling using machine learning (ML) algorithms when combined with multi-dimensional gait can be used to address this question [18]–[21]. Whilst this previous work points to the potential, it is limited to small sample sizes, limited gait characteristics and the risk of overfitting data due to high correlation with multiple variables derived from the same signal [9].

Because accelerometers can provide a large amount of gait characteristics, a comprehensive analysis on an adequately sized population of mild to moderate PD subjects is required to identify the optimal gait characteristics for use as state markers in PD. This study therefore aims: (i) to comprehensively quantify digital gait characteristics (spatiotemporal and signal-based) from a single accelerometer in people with mild to moderate PD, and (ii) to explore the best discriminative digital gait characteristics for optimal classification of PD. We hypothesize that a data driven approach where signal based characteristics combined with more typical spatiotemporal gait variables will be superior to quantify gait in PD and as a result, would contribute a feasible and objective method to aid the diagnosis of PD.

## II. Results

Table 1 shows demographics, cognitive and clinical characteristics of the participants. Compared to Controls (CLs), people with PD were of similar age, were shorter, weighed less and had significantly poorer global cognition. The average PD duration was 24 months from diagnosis at the time of gait assessment.

**TABLE 1.** Demographic and Clinical Characteristics

Characteristics	HC (n = 61) (Mean ± SD)	PD (n = 81) (Mean ± SD)	p
M/F (n)	34/27	53/28	0.873
Age (year)	69.99 ± 7.43	69.31 ± 10.44	0.663
Height (m)	1.69 ± 0.09	1.69 ± 0.08	0.731
Weight (Kg)	80.91 ± 15.22	78.15 ± 15.70	0.297
BMI (Kg/m²)	28.15 ± 4.12	27.34 ± 4.73	0.289
MOCA	27.37 ± 2.55	26.22 ± 3.26	**0.023**
NFOG		1.48 ± 4.23	
LEDD (mg/day)		397.73 ± 214.48	
Disease duration (Months)		24.11 ± 4.78	
Hoehn and Yahr (n)		HY I - 7	
		HY II - 60	
		HY III - 14	
MDS - UPDRS III		33.42 ± 10.33	
		(HY I-17.43 ± 4.83)	
		(HY II-34.18 ± 9.67)	
		(HY III-38.14 ± 7.54)	

M: Male; F: Female; BMI: Body mass index; MoCA: Montreal Cognitive Assessment; NFOG: New freezing of gait questionnaire; LEDD: Levodopa equivalent daily dose; MDS – UPDRS III: Movement Disorder Society - Unified Parkinson's Disease Rating Scale Part III. In bold significant p values (p < 0.05).

### A. Classification of PD

Six partial least square – discriminant analysis (PLS-DA) models were trained on the different sub-datasets (Table 2). Three to five latent variables (components) in all the PLS-DA models based on the predictive performance were enough to explain the total variance of independent variables (standard goodness of fit parameters (Q², R²X, and R²Y) provided in supplementary Figure S1).

**TABLE 2.** PLS-DA Classification Performance in PD From Different Combinations of Accelerometer Derived Gait Characteristics (Char) and Participant Demographic (DEM) Data

Characteristics	Confusion Matrix		Sensitivity	Specificity	Accuracy	
		CL	PD			
Spatiotemporal (ST)	CL	38	23	76.54	62.3	70.42
	PD	19	62			
Signal Char. (SC)	CL	51	10	90.12	83.61	87.32
	PD	8	73			
SC + ST	CL	50	11	90.12	81.97	86.62
	PD	8	73			
ST + DEM	CL	42	19	72.84	68.85	71.13
	PD	22	59			
SC + DEM	CL	52	9	90.12	85.25	88.03
	PD	8	73			
SC + ST+ DEM	CL	53	8	90.12	86.89	88.73
	PD	8	73			

Table 2 shows the classification performance. Overall signal based characteristics gave better classification performance (accuracy: 87.32%, sensitivity: 90.12%, specificity: 81.97%) compared to spatiotemporal characteristics alone (accuracy: 70.42%, sensitivity: 76.54%, specificity: 62.3%). By adding demographic (including MoCA) data to spatiotemporal and signal based characteristic models, the increase in classification performance was negligible (<2%), while specificity of the models improved by 2–4%. By combining the spatiotemporal characteristics to signal based characteristics the accuracy and specificity of the model decreased slightly (accuracy: 86.62%, sensitivity: 90.12%, specificity: 81.97%). However, this increased again when the demographics were added (accuracy: 88.73%, sensitivity: 90.12%, specificity: 86.89%) and is marginally better than the model trained on the signal based characteristics and demographics (accuracy: 88.03%, sensitivity: 90.12%, specificity: 85.25%).

**Figure 1.**
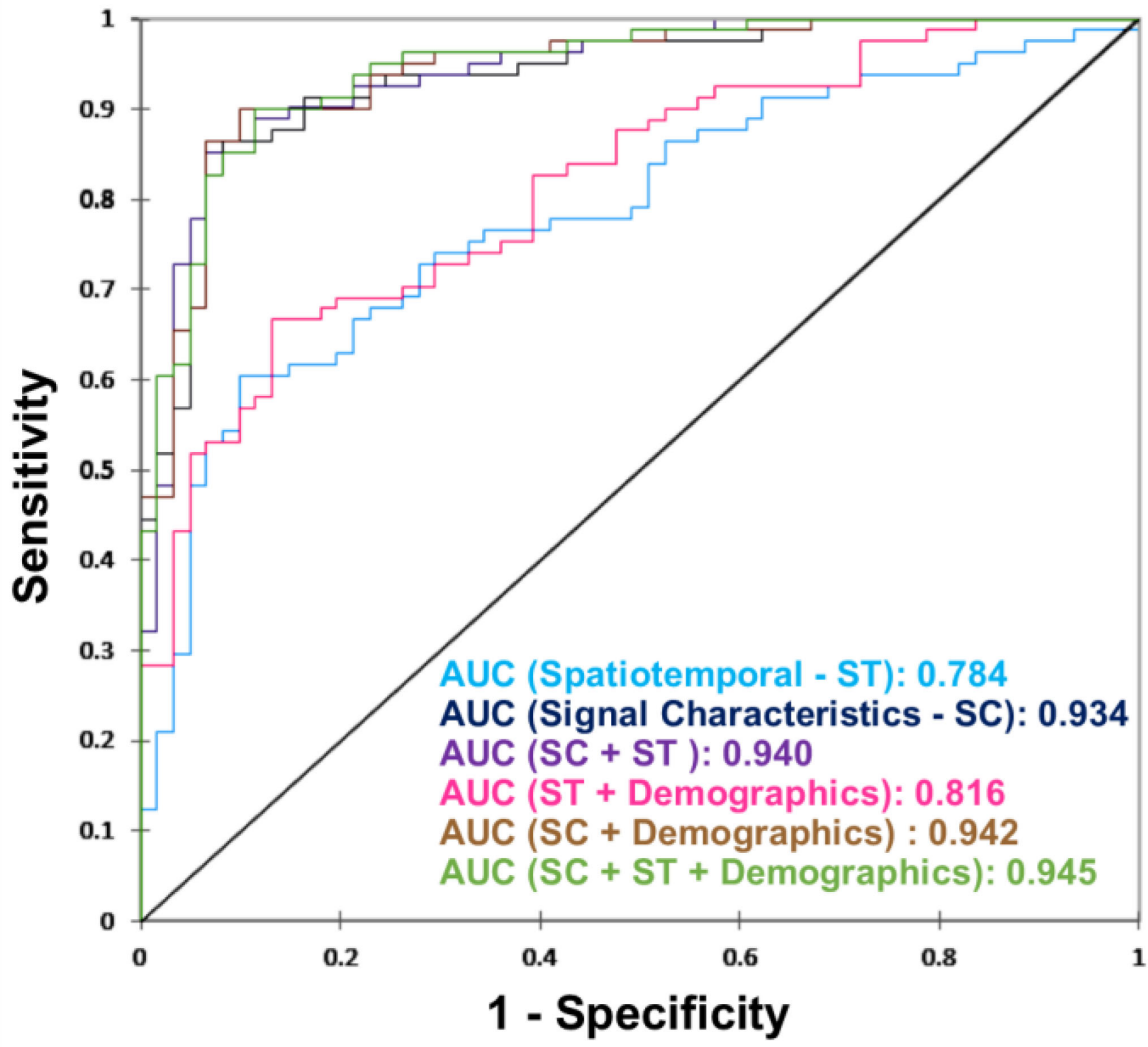
Receiver operating characteristics curve for each of the six classification models.

Figure 1 shows the area under the receiver operating characteristic curve (AUC) for all the six models. The AUC is higher for signal based characteristics as compared to spatiotemporal gait characteristics. The addition of demographics had negligible impact on models AUC.

### B. Important Characteristics in the Model

**Figure 2.**
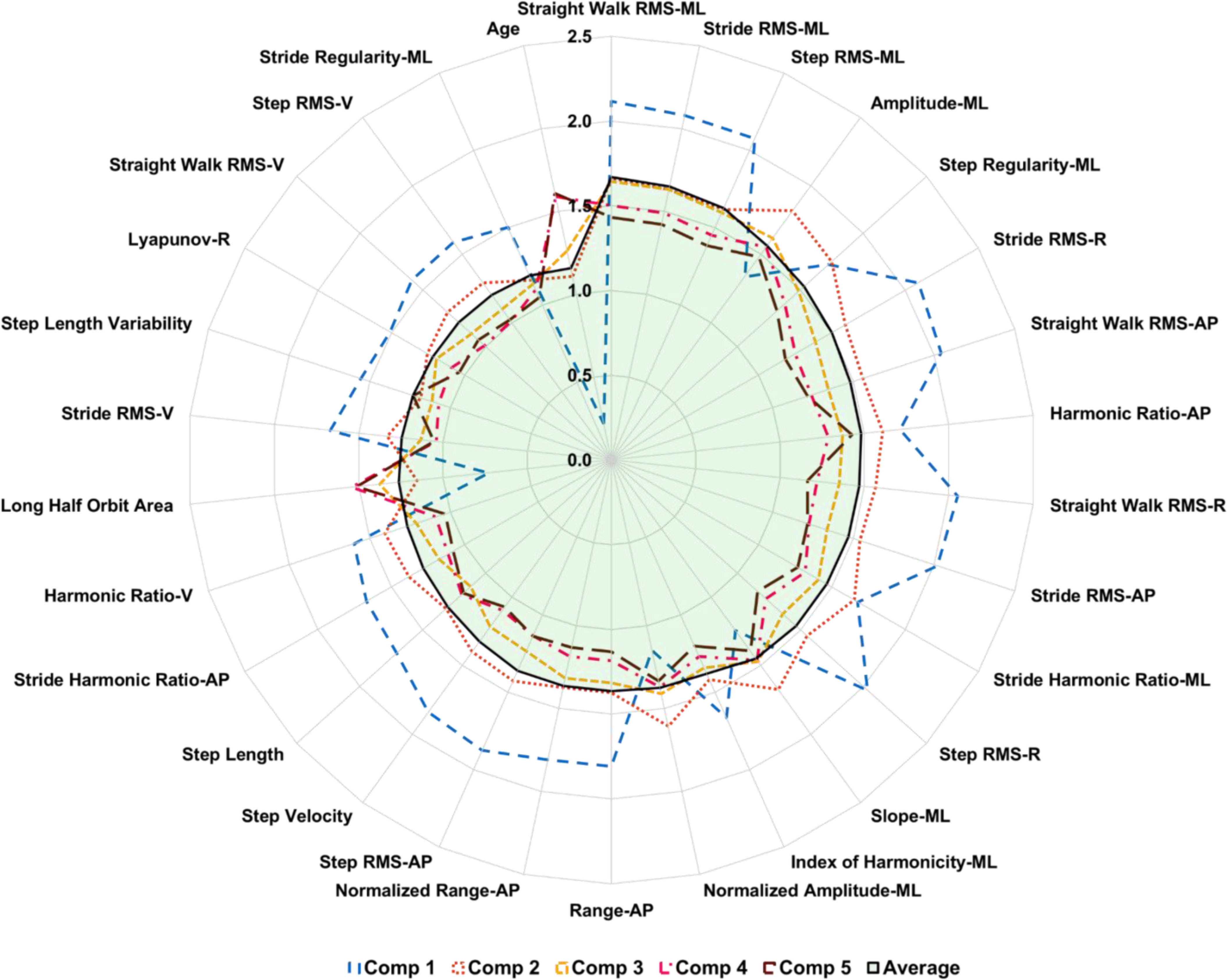
The importance of variables in the projection of the components (comp) on overall dataset. The further the line from 0 the more important the variable.

Figure 2 shows the characteristics with a Variable Importance in the Projection (VIP) [22] value of > = 1.5 in at least one of the PLS-DA model components trained on the overall data set. The VIP score for all variables (Table S1) and their definitions (Table S3) are provided in the supplementary material.

Characteristics are ranked based on the average VIP score of all the components in the model (Figure 2). Signal magnitude based measures such as root mean square (RMS) in all three directions (vertical (VT), mediolateral (ML) and anteroposterior (AP)) for each pass of straight walking, per stride and step were highly influential. Signal frequency domain measures such as the power spectral density (PSD) amplitude and slope of the signal in ML, harmonic ratio in AP & VT, index of harmonicity in ML, range of signal in AP and stride harmonic ratio in the ML & AP direction were important. Among signal regularity based characteristics, step and stride regularity in the ML direction were important. Spatiotemporal measures such as step velocity, step length, and step length variability were influential. Complexity of the signal, in the form of phase plot characteristic long half orbit area asymmetry and Lyapunov exponent derived from combined resultant axes from tri-axial accelerometer, were relevant. In this model, the age of the subjects was also important in the classification process.

### C. Statistical Significance of Important Characteristics Between PD and Cl

**Figure 3.**
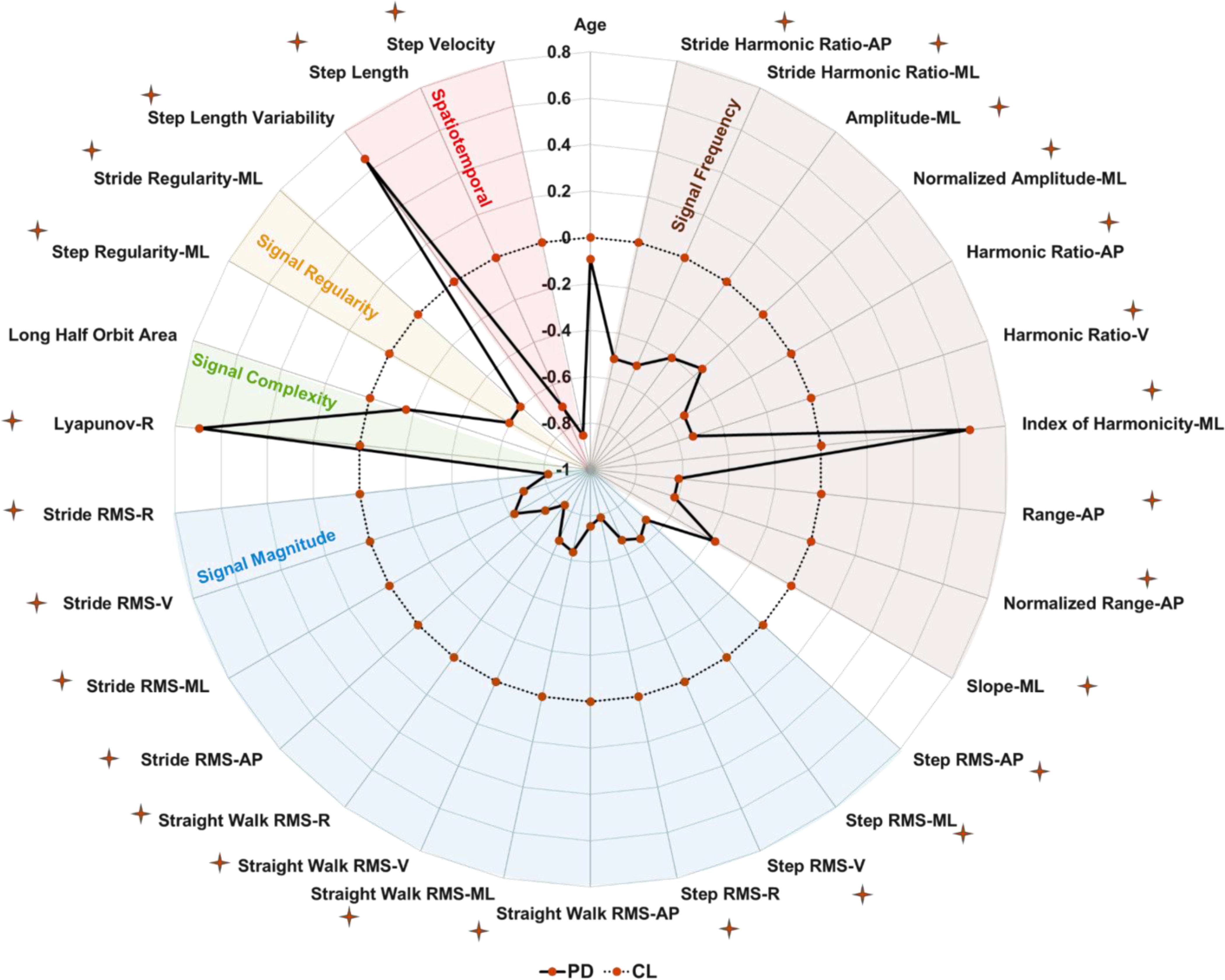
Statistical difference between people with PD (PD) and CL, characteristics are standardized into z-score, deviation from zero along the axis radiating from the center of the plot represents how many standard deviations the PD differ from CL (range: ±1 SD, z-score based on CL means and SDs), and star indicates p < 0.05.

On average, PD and CL groups walked 5 and 6 passes on the mat respectively, with an average of 42 steps for PD and 45 steps for CL. Compared to the CL group, PD had significantly lower signal magnitudes, signal frequency characteristics, regularity, complexity, step velocity and step length (Figure 3). In addition, PD had higher coefficient of variability in step length, Lyapunov exponent and signal index of harmonicity in PSD. The mean ± standard deviation of all 210 gait characteristics are available in the supplementary material (Table S2). Correlation analysis results between the gait characteristics are given in the supplementary material (Figure S2).

## III. Discussion

To the best of our knowledge, this is the most comprehensive study to quantify digital gait characteristics from a single tri-axial accelerometer and identify discriminative characteristics for optimal classification of early stages of PD. Two hundred and ten digital gait characteristics from spatiotemporal, signal magnitude, regularity, complexity and frequency domains were used in this study. A PLS-DA method, which can deal with multi-collinearity [22], [23], was used in the classification step. Based on the results, signal based characteristics (Acceleration RMS, PSD (amplitude, slope and range), harmonic ratio, index of harmonicity and regularity) added greater classification value compared to traditional spatiotemporal gait characteristics. This highlights the importance of extracting signal based digital characteristics to support the development of sensitive and objective pre-screening diagnostic tools, to support early identification of PD.

The variety of methodologies and data analysis techniques used across studies makes comparisons problematic due to inconsistent findings and variable classification accuracy [20], [24], [25]. Relative to past studies, the results here and the methodology used, show comparable or improved accuracy and better balance in sensitivity and specificity to classify people with PD. Assuming that the participants in this study are accurately diagnosed, the accuracy reported in this study is higher than that reported by movement disorder specialists [4]. These results were derived from pre-extracted signal based characteristics, tailored to assess multifaceted movement patterns required to quantify the synergistic movements seen in PD [14]. Deep learning methods such as convolutional neural networks (CNN), could be used for larger sample sizes to find the better classification accuracy from raw acceleration signals [18]. Potentially, these approaches could achieve favorable results from bigger datasets; however, the high accuracy reported in this study, combined with the ability to determine the key characteristics that contributed to it, is favorable from a clinical perspective. A lot of data driven approaches are based on a “black box” approach which may be difficult to fully understand and interpret. The adopted approach here provides interpretable information to describe how PD gait differs from older adult controls, therefore, unlike a black box method, it can provide targets for intervention.

The key characteristics that achieved VIP scores greater than 1.5 included a variety of traditionally adopted spatiotemporal information and acceleration signal-based characteristics. Spatiotemporal characteristics replicating variables from instrumented walkways have been predominantly assessed due to the advantage of increased interpretability [12], [26]. Due to their discrete nature, a drawback of these characteristics is that they solely quantify movements of the feet in the line of progression. For complex measures such as asymmetry and variability, which are highly prevalent in PD [26]–[28] even at the early stages [6], we argue that these gait characteristics are best quantified using information from multiple planes of motion [29], [30]. Here, the top five performing characteristics were from mediolateral signals, which due to being quantified at L5, are related to measures of stability/postural control during gait [31], [32]. We propose the additional information achieved through a comprehensive analysis of each component of the signal can better quantify these complex characteristics and is the reason for an improved classification accuracy. Previously when examining people with PD with the use of instrumented walkways, step width and its variability showed low correlation with other gait characteristics but was highly relevant for classification [33], [34]. To our knowledge, single accelerometers located on the lower back, cannot accurately quantify step width and the benefit of assessing it with the already included characteristics is unknown. Future research should aim to include step width, or equivalent proxy characteristics, so that it can contribute to an expected higher classification accuracy.

### A. Limitations and Future work

The PLS-DA model was trained and tested on a mild to moderate PD cohort, who had gait assessment within an average of 24 months from clinical diagnosis. We considered them at an early stage of PD and assumed that the participants were accurately diagnosed; all participants met the UK Brain Bank criteria for PD at the time of assessment. They therefore may not be generalizable to an older/younger group with greater/smaller disease severity and disease duration. All participants were on dopaminergic medication; although this reflects clinical practice, future studies should consider replicating these methods in a drug naïve cohort. Although it is presumed that state variables are good targets for the identification of trait markers, it is possible that findings from this study may not be generalizable for prodromal PD. A trained model should be tested on a diverse prodromal cohort followed longitudinally with diagnosis confirmed post mortem.

We used five domains to try to map the presented features, future work should also consider factor analysis approaches to determine gait models that includes independent domains to group gait variables [13]. Furthermore, future efforts should test if these variables are not only sensitive, but also specific to detect PD gait impairment, and should determine the generalizability of the results to other neurodegenerative diseases that present similar mobility impairment. Although accelerometers are proposed as a feasible tool, they are not currently adopted as part of PD diagnostics and substantial efforts are required to overcome the challenges preventing their potential adoption [35]. Wearable sensors are becoming smaller and combining multiple sensors in a single low price device is now possible [36]. It is plausible that at the time of potential clinical adoption, sensors such as gyroscopes and magnetometers could contribute towards a more accurate calculation of existing, or provide additional, movement based characteristics, such as turning quality. These characteristics may contribute to an improved classification accuracy. Research into their inclusion is therefore warranted. Gyroscopes would also improve the ability to detect straight line walking episodes in free living environments. This would allow us to assess gait within the participant's natural environment. Future work might focus on the replication of the analyses based on free living data.

### B. Applications/Clinical Implications

The objective nature of gait assessment with a wearable sensor, together with the practical advantages of its implementation to a clinical environment motivates its adoption. If this adoption becomes a reality, the comprehensive approach presented here performed better in terms of trade-off between sensitivity and specificity than previously proposed models and is built using clinically interpretable characteristics quantified with an accelerometer. The results from the current methodology provide evidence for a favorable approach to identify early movement diagnostic markers of PD. This improved accuracy is potentially a step in the right direction towards an approach that can aid predictions of specific disease progression and an understanding of the underlying mechanisms that underpin gait impairment in PD.

## IV. Conclusions

This study showed that a comprehensive approach that combined signal based characteristics with traditional measures of gait and participant demographic information, was optimal for the classification of the PD group. The results therefore show that, if using wearable sensors to provide potential state markers of PD, characteristics taken from the multiple signal based domains and planes of motion better highlight synergistic movements of people with PD. Additionally out of the 210 that were included, it highlighted which gait characteristics were the most capable to highlight these synergistic movements. It is hoped these results are a step towards the adoption of comprehensive approaches in future attempts to find the best movement based state markers at the early stages of PD. These approaches may be applicable for better classification at the prodromal stage or even between phenotypes where gait could be considered as a digital biomarker for PD.

## V. MATERIALS AND Methods

### A. Participants

Data from 81 people with PD and 61 CLs, collected as part of the “Incidence of Cognitive Impairment in Cohorts with Longitudinal Evaluation - GAIT” (ICICLE-GAIT) study, were used in this work [37]. The study was approved by the “Newcastle and North Tyneside research ethics committee” (REC No. 09/H0906/82). All the participants gave their written informed consent before participating in the study.

### B. Demographics and Clinical Measures

Demographic characteristics such as age, height and weight were recorded for all the subjects. Cognition was assessed with the Montreal Cognitive Assessment (MOCA) [38]. Freezing in gait was assessed with New Freezing of Gait (NFOG) questionnaire [39]. Levodopa equivalent daily dose (LEDD mg/day) was calculated according to defined criteria [40]. To assess PD motor severity, Hoehn & Yahr stage [41] and the Movement Disorder Society Unified Parkinson's Disease Rating Scale [42] (MDS-UPDRS) – Part III were used.

### C. Equipment

Participants wore a tri-axial accelerometer (Axivity AX3, dimensions: 23.0 × 32.5 × 7.6 mm, Sample frequency 100 Hz, Range: ± 8g), on the lower back (L5), affixed by double sided tape (BSN Medical Limited, Hull, U.K) [12]. An instrumented mat (Platinum model GAITRite: 7.0 × 0.6 m, Spatial accuracy: 1.27 cm, Temporal accuracy of 1 sample (240 Hz, ∼ 4.17 ms)) was used for accurate segmentation and identification of walking.

### D. Testing Protocols and Data Segmentation

**Figure 4.**
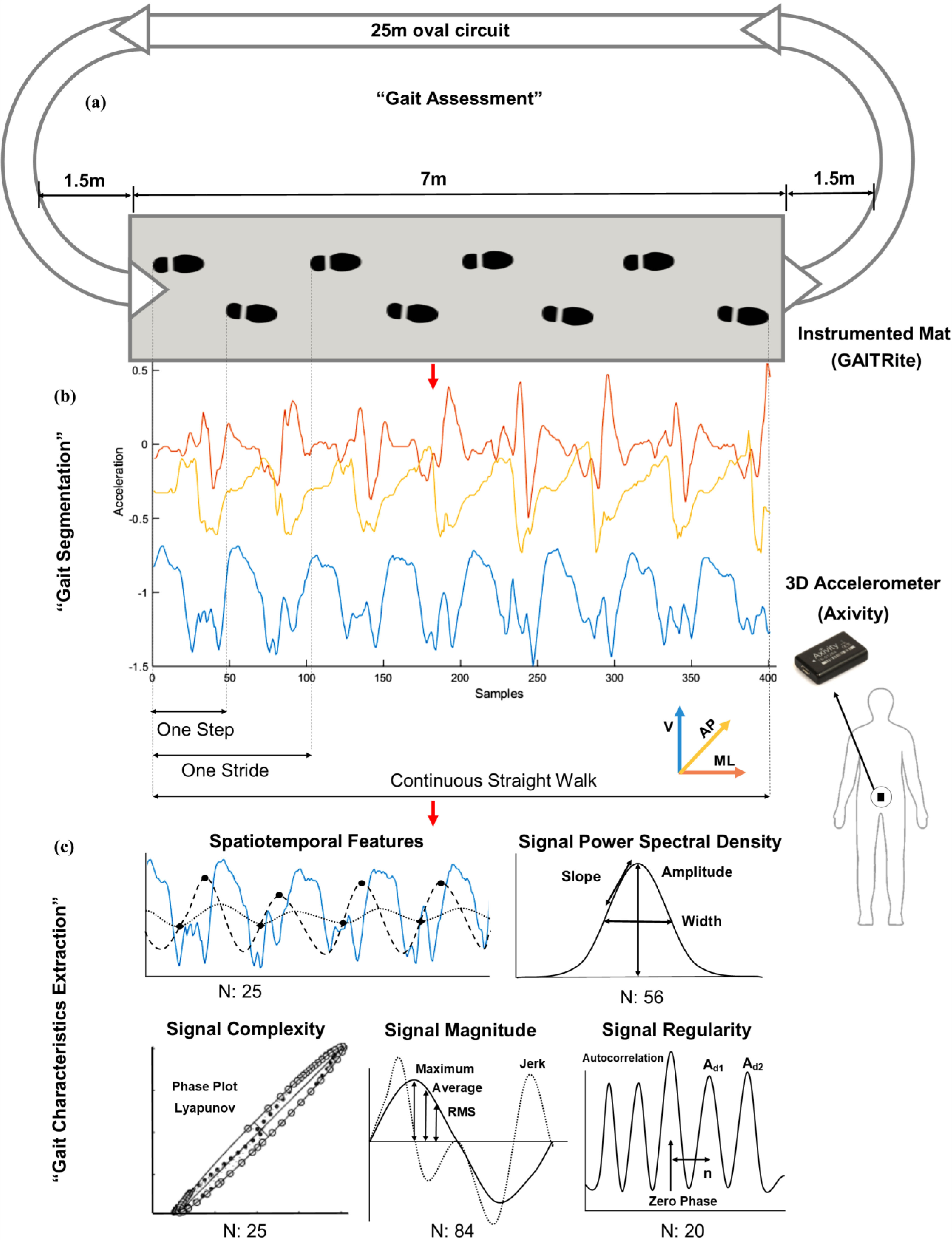
Process flow for quantification of gait characteristics: (a) Gait assessment in the lab, (b) Accelerometery signal segmentation based on GAITRite timing for each pass, stride and step, (c) Extraction of gait characteristics

PD participants were assessed one hour after dopaminergic medication intake. Participants walked at their preferred walking speed for two minutes continuously over a 25 m oval circuit (Figure 4-(a)). Axivity was synchronized with the real-time clock of GAITRite. Straight walks with each pass on mat, strides, and steps were automatically segmented in Matlab based on the heel strike and toe-off timings from the GAITRite mat (Figure 4-(b)).

### E. Gait Characteristics Extraction

Along with spatiotemporal characteristics, various signal-based gait characteristics were extracted (defined in the supplementary material (Table S3)). Depending on the characteristic, segmentation of data on a step-by-step, stride-by-stride or multiple consecutive strides basis was required. Two hundred and ten gait characteristics (spatiotemporal and signal-based) were extracted and divided into five different domains (spatiotemporal, frequency based, signal magnitude, signal regularity, and signal complexity) as shown in Figure 4-(c).

In the spatiotemporal domain (N: 25 characteristics), 14 gait characteristics were extracted based on previous work [12]. Cadence, and the coefficient of variability for both step and strides characteristics was added. In the signal power spectral density domain we extracted frequency based characteristics (N: 56) including amplitude, width, slope, and range of the dominant peak from the power spectral density [43], harmonic ratio [44], and index of harmonicity [45]. In the signal regularity domain (N: 20), various outputs from deriving the step and stride regularity from performing autocorrelation and also, the gait symmetry index were quantified [46], [47]. From the signal magnitude domain (N: 84), root mean square, jerk, jerk ratio, maximum and minimum values were extracted for each step, stride, and straight walk from each axis of the signal [14]. In the complexity domain (N: 25), we included geometrical characteristics extracted based on the shapes in the phase plots [48] along with Lyapunov exponents [49].

### F. Classification Modeling and Variable Importance

Partial Least Square (PLS) regression [50] combined with discriminant analysis (PLS-DA) [23] was used to handle 216 independent characteristics (including gait, demographics and clinical information) to classify two dependent variables (people with PD & CL) from a relatively low number of subjects (N = 142). The motivation to use this method and details about it are given in the supplementary material S1. A separate model for each selection of independent characteristics was built for classification of PD. The number of components for the model was determined on the cross-validation performance in PLS-DA. The quality of each predictive model based on the number of components, was determined by the cumulated index Q², which assesses global fitness (predictive accuracy). Its value should be greater than 0, with values close to 1 being ideal for identifying the most relevant components in the model. Similarly, to determine the explanatory power of the components for the independent and dependent variables, cumulative index of R²X and R²Y were used respectively to determine the quality of the model. Ideally, these indexes should be greater than 0 and close to 1 for each component to be included in the model. The importance of each independent variable in the model was determined based on the projection (VIP) score, which shows the importance of the explanatory variables for building the model components [22]. The VIP score was used to identify the variables that were moderately (0.8 < VIP < 1) or highly influential (VIP > 1) in the model [22]. Independent t-tests were performed on these identified variables to evaluate the difference between people with PD and CL. Pearson's correlation analysis was also performed to check dependency among the important gait characteristics.

## Supplementary Materials

S1: Motivation to use PLS-DA and method detail. Figure S1: PLS-DA models quality based on the number of components. Table S1: Variable importance in the projection of the components in PLS-DA. Table S2: Difference between people with PD and CL based on independent sample t-test. Table S3: Definition of gait characteristics used in the study.


