# Supplementary material for: Accelerometry-Based Digital Gait Characteristics for Classification of Parkinson's Disease: What Counts?
Source: IEEE Open J Eng Med Biol. 2020 Feb 14;1:65–73. doi: 10.1109/OJEMB.2020.2966295 (PMC8979631; doi:10.1109/OJEMB.2020.2966295)
Supplement: Supplementary file 1 [file supp1-2966295.pdf]

Supplementary Materials

Accelerometry-based digital gait characteristics for classification of Parkinson's disease: what counts?

Rana Zia Ur Rehman†, Christopher Buckley†, Maria Encarna Micó-Amigo, Cameron Kirk, Michael Dunne-Willows, Claudia Mazzà, Jian Qing Shi, Lisa Alcock, Lynn Rochester, and Silvia Del Din* Member, IEEE

S1: Motivation to use the PLS-DA

Typically, to achieve data reduction, principal component analysis (PCA) is performed prior to classification [1]. However, while PCA only takes into account independent variables and generates new components containing high variance without losing information, its major drawback is the potential loss of important information about how independent variables (e.g. gait characteristics) are related to the dependent variables (e.g. groups being classified) [2]. Other techniques like Partial least square discriminant analysis (PLS-DA) can help to tackle this downside as it can deal with situations in which we have a higher number of multi-collinear independent variables compared to the number of subjects in the study and their relation with dependent variables (e.g. groups to classify (PD vs. CL)) [3].

Method (PLS-DA) Detail

PLS-DA is used for the data reduction and to classify the subjects with Parkinson's disease from controls based on the latent variables (capturing high variance) used as training data in discriminant analysis for classification. This method can provide the contribution of the independent variables (gait characteristics) in the classification modeling based on the variable importance in the projection (VIP) [4], which is calculated with the following equation 1.

$$VIP_j = \sqrt{p\sum_{k=1}^{N}\frac{\left[SS_k\left(\frac{w_{kj}}{\left||w_k|\right|^2}\right)\right]}{\sum_{k=1}^{N}(SS)_k}} \qquad (1)$$

p is a total number of gait characteristics in the model. N is the number of latent variables in the PLS-DA model. ss_k is the sum of variance of the latent variable, w_{kj} quantify the contribution of gait characteristics j according to the kth latent variable. w_k is the contribution of the kth latent variable. If the VIP is above 1 then the particular gait characteristic is highly influential in the model and higher VIP value indicates the higher contribution of the gait characteristic.

The model quality is based on the standard goodness of fit indices such as Q2, R2X and R2Y (Figure S1). Number of components (latent variables) in the PLS-DA are based on these indices. Q2 explains the predictive capability of the model, and with its value the suitable number of components in the PLS-DA can be identified. If the value of Q2 is higher than 0 then model prediction capability is better, if its value is below 0 then model performance will be poor. Therefore, based on its value we selected the number of components in the PLS-DA. Other indices, such as R2X and R2Y explain the captured variance by independent and dependent variables respectively.

MODEL QUALITY

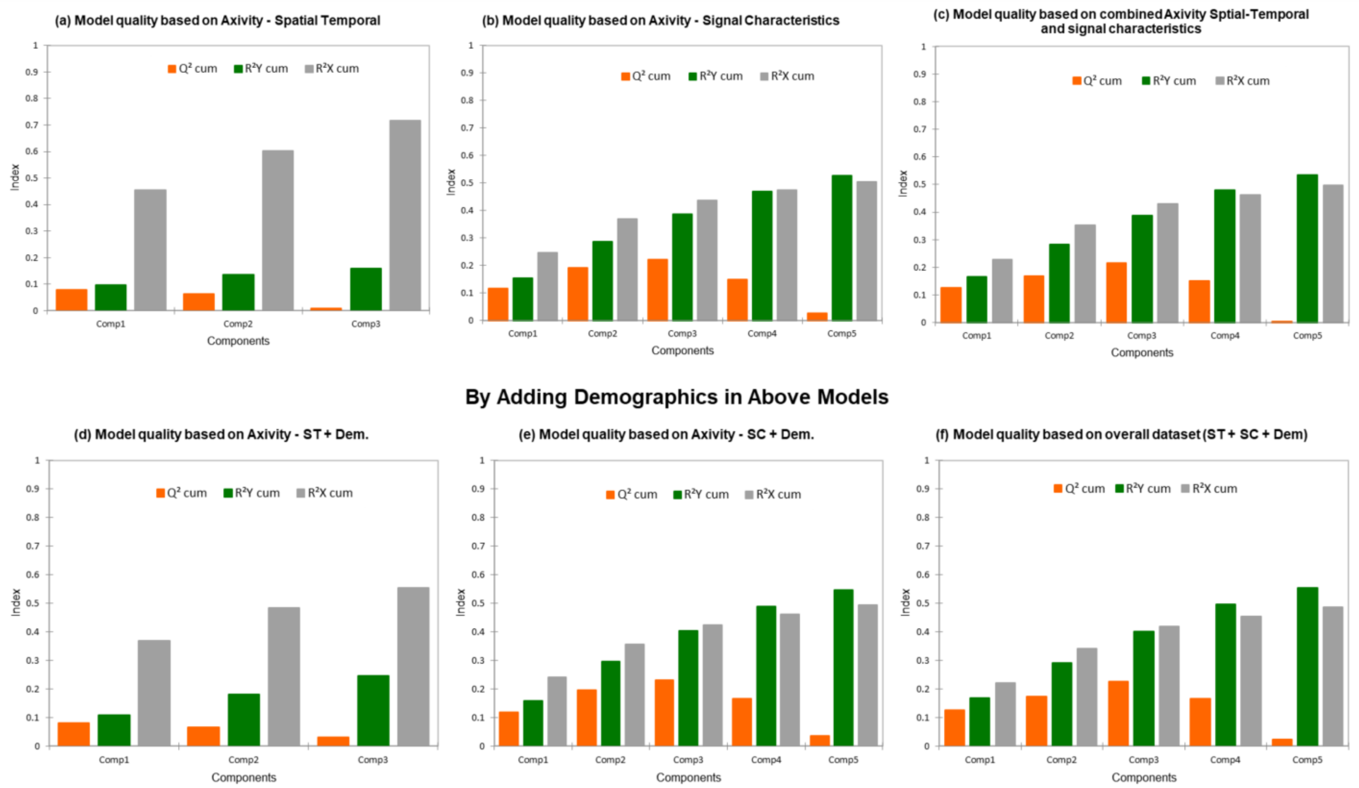

Fig. S1. PLS-DA models quality based on the number of components

TABLE S1.
VARIABLES' IMPORTANCE IN THE PROJECTION OF THE COMPONENTS IN PLS-DA

Domain	Variable	Comp1	Comp 2	Comp 3	Comp 4	Comp 5	Average
Spatial Temporal	Step Velocity	1.840	1.398	1.217	1.096	1.071	1.325
	Step Length	1.705	1.313	1.119	1.188	1.175	1.300
	Step Length Coefficient of Variation (CV)	1.556	1.183	1.113	1.073	1.231	1.231
	Swing Time Variability	1.463	1.118	1.232	1.171	1.126	1.222
	Cadence	1.010	1.120	1.144	1.437	1.360	1.214
	Step Velocity (CV)	1.499	1.141	1.136	1.066	1.020	1.172
	Step Time Variability	1.362	1.085	1.156	1.042	1.071	1.143
	Swing Time (CV)	1.312	1.004	1.144	1.137	1.101	1.139
	Stride Time Variability	1.372	1.068	1.106	0.996	1.043	1.117
	Stride Time (CV)	1.324	1.029	1.089	1.014	1.060	1.103
	Step Length Variability	1.382	1.051	0.965	0.966	1.119	1.097
	Step Time (CV)	1.248	1.003	1.110	1.028	1.059	1.09
	Stance Time Variability	1.056	0.818	1.231	1.164	1.135	1.081
	Stance Time (CV)	0.937	0.724	1.189	1.153	1.128	1.026
	Stride Time	1.093	0.831	0.774	1.130	1.091	0.984
	Swing Time	1.267	0.963	0.866	0.933	0.883	0.982

	Step Time	1.089	0.828	0.772	1.131	1.091	0.982
	Swing Time Asymmetry	0.728	0.571	1.160	1.054	1.002	0.903
	Stance Time	0.852	0.668	0.667	1.185	1.129	0.900
	Step Length Asymmetry	0.973	0.742	0.827	0.751	1.029	0.864
	Step Time Asymmetry	0.948	0.786	0.869	0.786	0.771	0.832
	Step Velocity Variability	0.908	0.690	0.761	0.901	0.857	0.823
	Stance Time Asymmetry	0.574	0.487	1.091	0.993	0.939	0.817
	Stride Time Asymmetry	0.451	0.347	0.713	1.130	1.069	0.742
	Step Velocity Asymmetry	0.331	0.252	0.593	0.547	0.537	0.452
Signals power spectral density	Power spectral density (PSD) Amplitude (ML)	1.337	1.817	1.619	1.553	1.479	1.561
	Harmonic Ratio (AP)	1.712	1.607	1.370	1.286	1.426	1.480
	Slope of dominant peak of PSD (ML)	1.243	1.673	1.470	1.460	1.393	1.448
	Index of Harmonicity (ML)	1.666	1.418	1.342	1.270	1.201	1.379
	Normalized amplitude of dominant peak of PSD (ML)	1.150	1.608	1.411	1.372	1.334	1.375
	Range of dominant peak of PSD (AP)	1.808	1.374	1.317	1.186	1.133	1.364
	PSD Normalized Range (AP)	1.808	1.374	1.317	1.186	1.133	1.364
	PSD Amplitude (R)	1.486	1.405	1.300	1.190	1.190	1.314
	Slope of dominant peak of PSD (R)	1.469	1.376	1.296	1.184	1.181	1.301
	PSD Normalized Slope (ML)	1.065	1.475	1.279	1.296	1.263	1.276
	Harmonic Ratio (V)	1.596	1.405	1.211	1.091	1.033	1.267
	Harmonic Ratio (ML)	1.394	1.388	1.191	1.104	1.127	1.241
	PSD Amplitude (V)	1.251	1.181	1.114	1.145	1.142	1.167
	Range of dominant peak of PSD (ML)	1.435	1.098	1.194	1.080	1.021	1.166
	PSD Normalized Range (ML)	1.435	1.098	1.194	1.080	1.021	1.166
	Slope of dominant peak of PSD (V)	1.236	1.152	1.110	1.139	1.133	1.154
	Normalized amplitude of dominant peak of PSD (R)	1.260	1.111	1.172	1.087	1.078	1.142
	PSD Mean Power (V)	0.889	1.204	1.271	1.151	1.176	1.138
	PSD Integrated Power (V)	0.889	1.204	1.271	1.151	1.176	1.138
	Range of dominant peak of PSD (R)	1.262	1.089	1.134	1.121	1.061	1.133
	PSD Normalized Range (R)	1.262	1.089	1.134	1.121	1.061	1.133
	PSD Normalized Slope (R)	1.229	1.073	1.168	1.082	1.069	1.124
	Index of Harmonicity (AP)	1.083	0.913	1.112	1.241	1.195	1.109
	Index of Harmonicity (R)	1.360	1.104	1.097	0.990	0.937	1.098
	Range of dominant peak of PSD (V)	1.161	1.042	1.089	1.113	1.071	1.095
	PSD Normalized Range (V)	1.161	1.042	1.089	1.113	1.071	1.095
	Harmonic Ratio (R)	1.312	1.078	1.014	0.987	0.968	1.072
	Normalized amplitude of dominant peak of PSD (V)	1.026	0.894	1.013	1.070	1.064	1.013
	PSD Normalized Slope (V)	1.000	0.860	1.015	1.065	1.056	0.999
	PSD Mean Power (R)	0.714	1.010	1.077	1.020	1.096	0.983
	PSD Integrated Power (R)	0.714	1.010	1.077	1.020	1.096	0.983
	Dominant Frequency (V)	0.321	0.598	1.169	1.064	1.024	0.835

	Index of Harmonicity (V)	0.877	0.676	0.834	0.801	0.758	0.789
	PSD Mean Power (ML)	0.555	0.651	0.748	0.755	1.065	0.755
	PSD Integrated Power (ML)	0.555	0.651	0.748	0.755	1.065	0.755
	Width of dominant peak of PSD (V)	0.073	0.441	1.021	0.970	1.095	0.720
	PSD Normalized Width (V)	0.073	0.441	1.021	0.970	1.095	0.720
	PSD Amplitude (AP)	0.460	0.558	0.824	0.847	0.860	0.710
	Slope of dominant peak of PSD (AP)	0.432	0.558	0.829	0.842	0.861	0.704
	PSD Normalized amplitude (AP)	0.380	0.493	0.859	0.806	0.853	0.678
	PSD Normalized Slope (AP)	0.352	0.494	0.857	0.799	0.851	0.670
	PSD Median Power (ML)	0.01	0.235	0.518	1.312	1.246	0.664
	PSD Median Power (AP)	0.087	0.427	0.649	1.080	1.024	0.653
	Width of dominant peak of PSD (R)	0.148	0.437	0.816	0.790	0.98	0.634
	PSD Normalized Width (R)	0.148	0.437	0.816	0.790	0.98	0.634
	Width of dominant peak of PSD ML)	0.154	0.299	0.733	0.996	0.976	0.632
	PSD Normalized Width (ML)	0.154	0.299	0.733	0.996	0.976	0.632
	PSD Median Power (R)	0.120	0.370	0.682	0.965	0.928	0.613
	Width of dominant peak of PSD (AP)	0.559	0.582	0.538	0.632	0.674	0.597
	PSD Normalized Width (AP)	0.559	0.582	0.538	0.632	0.674	0.597
	PSD Mean Power (AP)	0.463	0.352	0.428	0.843	0.862	0.590
	PSD Integrated Power (AP)	0.463	0.352	0.428	0.843	0.862	0.590
	PSD Median Power (V)	0.124	0.493	0.719	0.777	0.737	0.570
	Dominant Frequency (ML)	0.364	0.471	0.401	0.389	0.763	0.477
	Dominant Frequency (R)	0.017	0.103	0.688	0.707	0.675	0.438
	Dominant Frequency (AP)	0.239	0.377	0.386	0.372	0.36	0.347
Signal Magnitude	Root mean square (RMS) of Straight Walk (ML)	2.115	1.663	1.642	1.500	1.431	1.670
	RMS Stride (ML)	2.078	1.632	1.63	1.489	1.420	1.650
	RMS Step (ML)	2.076	1.619	1.598	1.451	1.385	1.626
	RMS Stride (R)	2.092	1.592	1.389	1.251	1.186	1.502
	RMS of Straight Walk (AP)	2.044	1.559	1.345	1.241	1.216	1.481
	RMS Straight Walk (R)	2.053	1.567	1.35	1.219	1.162	1.470
	RMS Stride (AP)	2.021	1.540	1.337	1.225	1.216	1.468
	RMS Step (R)	2.032	1.551	1.359	1.224	1.159	1.465
	RMS Step (AP)	1.877	1.427	1.237	1.137	1.139	1.363
	RMS Stride (V)	1.669	1.331	1.132	1.039	1.053	1.245
	RMS Straight Walk (V)	1.585	1.302	1.113	1.009	1.057	1.214
	RMS Step (V)	1.589	1.293	1.100	1.009	1.022	1.202
	RMS Ratio Step (ML)	0.659	0.758	0.947	1.092	1.035	0.898
	RMS Ratio Straight Walk (V)	0.205	0.716	0.843	1.130	1.297	0.838
	RMS Ratio Step (V)	0.417	0.701	0.742	1.098	1.197	0.831
	RMS Ratio Straight Walk (ML)	0.323	0.650	0.983	1.108	1.068	0.826
	RMS Ratio Stride (ML)	0.331	0.582	0.873	1.105	1.050	0.788

	RMS Ratio Stride (V)	0.177	0.634	0.719	1.129	1.246	0.781
	RMS Ratio Step (AP)	0.379	0.293	0.340	0.535	0.917	0.493
	RMS Ratio Stride (AP)	0.275	0.318	0.302	0.487	1.011	0.478
	RMS Ratio Straight Walk (AP)	0.182	0.360	0.313	0.470	1.027	0.471
Signal Regularity	Step Regularity (ML)	1.722	1.747	1.487	1.376	1.314	1.529
	Stride Harmonic Ratio (ML)	1.678	1.652	1.411	1.321	1.269	1.466
	Stride Harmonic Ratio (AP)	1.671	1.381	1.174	1.112	1.078	1.283
	Stride Regularity (ML)	1.504	1.163	1.137	1.100	1.049	1.190
	Step Regularity (V)	0.969	0.760	1.295	1.176	1.158	1.072
	Stride Harmonic Ratio (V)	1.240	1.014	0.996	0.897	0.903	1.010
	Stride Regularity (V)	0.858	0.663	1.244	1.149	1.094	1.002
	Symmetry Autocorrelation Difference (ML)	0.458	1.144	1.112	1.041	1.036	0.958
	Stride Regularity (AP)	0.946	0.719	1.105	1.019	0.979	0.954
	Step Regularity (AP)	0.800	0.638	1.138	1.038	1.009	0.925
	Step Regularity (R)	1.072	0.814	0.843	0.904	0.870	0.901
	Stride Regularity (R)	0.986	0.789	0.799	0.772	0.731	0.815
	Symmetry Autocorrelation Ratio (ML)	0.141	0.898	1.068	0.974	0.946	0.805
	Gait Symmetry Index	0.266	0.293	1.054	1.017	0.964	0.719
	Symmetry Autocorrelation Difference (V)	0.789	0.600	0.668	0.652	0.631	0.668
	Symmetry Autocorrelation Difference (AP)	0.299	0.393	0.774	0.717	0.739	0.584
	Symmetry Autocorrelation Ratio (V)	0.542	0.444	0.657	0.596	0.673	0.583
	Symmetry Autocorrelation Ratio (AP)	0.361	0.359	0.704	0.635	0.620	0.536
	Symmetry Autocorrelation Difference (R)	0.407	0.318	0.422	0.543	0.546	0.447
	Symmetry Autocorrelation Ratio (R)	0.435	0.384	0.386	0.480	0.475	0.432
Signal Complexity	Jerk RMS of Straight walk (AP)	1.353	1.213	1.051	1.083	1.024	1.145
	Jerk RMS of Straight walk (R)	1.012	1.336	1.144	1.052	0.997	1.108
	Jerk RMS of Straight walk (ML)	1.137	1.114	1.002	0.924	0.881	1.011
	Jerk RMS of Straight walk (V)	1.021	1.318	1.128	1.016	0.967	1.090
	Jerk RMS of Stride (AP)	1.360	1.204	1.053	1.080	1.022	1.144
	Jerk RMS of Stride (R)	1.053	1.308	1.128	1.035	0.983	1.102
	Jerk RMS of Stride (ML)	1.134	1.096	0.999	0.925	0.881	1.007
	Jerk RMS of Stride (V)	1.042	1.302	1.120	1.008	0.958	1.086
	Jerk RMS of Step (AP)	1.355	1.228	1.058	1.090	1.030	1.152
	Jerk RMS of Step (R)	1.021	1.346	1.148	1.066	1.009	1.118
	Jerk RMS of Step (ML)	1.132	1.107	0.999	0.918	0.871	1.005
	Jerk RMS of Step (V)	0.994	1.337	1.140	1.027	0.981	1.096
	Jerk Mean Ratio of Straight Walk (AP)	0.783	0.844	0.827	0.857	0.916	0.846
	Jerk Mean Ratio of Straight Walk (ML)	0.636	0.832	0.816	0.762	0.724	0.754
	Jerk Mean Ratio of Straight Walk (V)	0.716	0.702	0.608	0.550	0.556	0.626
	Jerk Mean Ratio of Stride (AP)	0.341	0.373	0.463	0.528	0.512	0.443

Jerk Mean Ratio of Stride (ML)	0.018	0.037	0.152	0.448	0.536	0.238
Jerk Mean Ratio of Stride (V)	0.260	0.198	0.222	0.204	0.422	0.261
Jerk Mean Ratio of Step (AP)	0.441	0.437	0.376	0.378	0.545	0.435
Jerk Mean Ratio of Step (ML)	0.370	0.335	0.285	0.355	0.496	0.368
Jerk Mean Ratio of Step (V)	0.410	0.401	0.346	0.363	0.606	0.425
Jerk Mean Log Ratio of Straight Walk (AP)	0.114	0.466	0.411	0.973	0.934	0.580
Jerk Mean Log Ratio of Straight Walk (ML)	0.183	0.287	0.247	0.569	0.568	0.371
Jerk Mean Log Ratio of Stride (AP)	0.569	0.670	0.577	0.523	0.495	0.567
Jerk Mean Log Ratio of Stride (ML)	0.569	0.670	0.577	0.523	0.495	0.567
Jerk Mean Log Ratio of Step (AP)	0.569	0.670	0.577	0.523	0.495	0.567
Jerk Mean Log Ratio of Step (ML)	0.569	0.670	0.577	0.523	0.495	0.567
Jerk Max of Straight Walk (AP)	0.832	0.847	0.867	0.829	0.791	0.833
Jerk Max of Straight Walk (R)	0.483	1.323	1.152	1.041	1.036	1.007
Jerk Max of Straight Walk (ML)	0.672	1.036	0.970	0.879	0.834	0.878
Jerk Max of Straight Walk (V)	0.778	1.091	1.068	1.102	1.128	1.033
Jerk Max of Stride (AP)	0.989	0.902	0.925	0.860	0.815	0.898
Jerk Max of Stride (R)	0.585	1.312	1.143	1.031	1.031	1.020
Jerk Max of Stride (ML)	0.789	1.052	0.979	0.888	0.841	0.91
Jerk Max of Stride(V)	0.841	1.123	1.079	1.085	1.096	1.045
Jerk Max of Step (AP)	1.096	0.990	0.958	0.881	0.841	0.953
Jerk Max of Step (R)	0.598	1.379	1.181	1.064	1.045	1.053
Jerk Max of Step (ML)	0.868	1.064	0.985	0.888	0.841	0.929
Jerk Max of Step (V)	0.672	1.276	1.107	1.021	0.977	1.011
Jerk Min of Straight Walk (AP)	0.546	1.236	1.052	1.317	1.257	1.081
Jerk Min of Straight Walk (R)	0.615	1.222	1.104	1.014	1.009	0.993
Jerk Min of Straight Walk (ML)	0.516	1.124	0.969	0.921	0.919	0.890
Jerk Min of Straight Walk (V)	0.572	1.214	1.109	1.063	1.023	0.996
Jerk Min of Stride (AP)	0.698	1.256	1.072	1.343	1.282	1.130
Jerk Min of Stride (R)	0.635	1.254	1.119	1.019	1.006	1.007
Jerk Min of Stride (ML)	0.740	1.063	0.953	0.873	0.846	0.895
Jerk Min of Stride (V)	0.656	1.217	1.100	1.047	0.997	1.004
Jerk Min of Step (AP)	0.836	1.278	1.097	1.304	1.239	1.151
Jerk Min of Step (R)	0.558	1.423	1.216	1.101	1.047	1.069
Jerk Min of Step (ML)	0.825	1.056	0.970	0.874	0.830	0.911
Jerk Min of Step (V)	0.694	1.263	1.114	1.058	1.012	1.028
Jerk Range of Straight Walk (AP)	0.721	1.098	0.958	1.091	1.032	0.980
Jerk Range of Straight Walk (R)	0.560	1.307	1.156	1.051	1.046	1.024
Jerk Range of Straight Walk (ML)	0.609	1.109	0.986	0.912	0.870	0.897
Jerk Range of Straight Walk (V)	0.705	1.187	1.121	1.114	1.103	1.046

Jerk Range of Stride (AP)	0.887	1.134	0.984	1.101	1.044	1.030
Jerk Range of Stride (R)	0.623	1.314	1.157	1.047	1.041	1.036
Jerk Range of Stride (ML)	0.781	1.081	0.986	0.898	0.852	0.920
Jerk Range of Stride (V)	0.779	1.202	1.120	1.096	1.069	1.053
Jerk Range of Step(AP)	1.007	1.199	1.027	1.105	1.046	1.077
Jerk Range of Step (R)	0.589	1.423	1.217	1.098	1.059	1.077
Jerk Range of Step (ML)	0.857	1.073	0.989	0.891	0.843	0.931
Jerk Range of Step (V)	0.703	1.308	1.144	1.069	1.022	1.049
Lyapunov RC (AP)	0.104	0.155	0.393	0.759	0.857	0.454
Lyapunov RC (R)	0.446	0.41	0.554	0.541	0.646	0.519
Lyapunov RC (ML)	0.025	0.232	0.614	0.643	0.636	0.430
Lyapunov RC (V)	0.457	0.786	0.844	1.109	1.129	0.865
Lyapunov W (AP)	1.156	1.026	1.051	0.948	0.905	1.017
Lyapunov W (R)	1.508	1.255	1.195	1.090	1.037	1.217
Lyapunov W (ML)	0.955	0.74	0.716	1.392	1.324	1.026
Lyapunov W (V)	1.218	0.962	1.146	1.037	1.095	1.092
Full Orbit Eccentricity	1.366	1.04	1.175	1.073	1.031	1.137
Relative Orbit Inclination	0.715	0.611	0.790	0.829	0.894	0.768
Full Orbit Area	0.309	0.387	0.645	0.609	0.849	0.560
Full Orbit Minor axis SD	0.542	0.502	0.428	0.673	0.882	0.605
Full Orbit Major axis SD	0.190	0.628	0.910	0.826	0.786	0.668
Short Half Orbit Eccentricity Asymmetry	1.166	0.923	1.211	1.257	1.334	1.178
Short Half Orbit Segment angle	1.350	1.031	1.050	0.951	0.929	1.062
Short Half Orbit Area Asymmetry	0.303	0.354	0.307	0.288	0.308	0.312
Long Half Orbit Eccentricity Asymmetry	1.022	0.800	0.820	0.741	0.708	0.818
Long Half Orbit Segment Angle	0.862	0.656	0.863	0.800	0.757	0.787
Long Half Orbit Area Asymmetry	0.303	0.354	0.307	0.288	0.308	0.312
Intra-Step Correlation	1.468	1.121	1.238	1.128	1.074	1.206

TABLE S2.
DIFFERENCE BETWEEN PEOPLE WITH PD AND CL BASED ON THE INDEPENDENT SAMPLES T-TEST

Domain	Variable	CL (n=61) Mean ± SD	PD (n=81) Mean ± SD	Normality test - Mann Whitney	Non-Parametric t-test	Parametric t-test

Spatial Temporal	Step Length	0.708 ± 0.077	0.653 ± 0.097	0.001	0.001	0.001
	Step Velocity	1.324 ± 0.164	1.184 ± 0.238	< 0.001	0.001	0.001
	Step Time	0.539 ± 0.045	0.568 ± 0.094	< 0.001	0.033	0.027
	Stride Time	1.078 ± 0.090	1.138 ± 0.192	< 0.001	0.034	0.026
	Swing Time	0.384 ± 0.041	0.404 ± 0.047	0.001	0.015	0.010
	Stance Time	0.696 ± 0.058	0.722 ± 0.106	< 0.001	0.141	0.084
	Cadence	67.161 ± 7.902	70.157 ± 8.987	0.287	0.037	0.040
	Step Length Variability	0.044 ± 0.017	0.054 ± 0.021	0.001	0.002	0.005
	Step Velocity Variability	0.097 ± 0.046	0.112 ± 0.047	< 0.001	0.017	0.066
	Step Time Variability	0.024 ± 0.015	0.036 ± 0.030	< 0.001	0.002	0.005
	Stride Time Variability	0.019 ± 0.007	0.032 ± 0.035	< 0.001	0.003	0.005
	Swing Time Variability	0.022 ± 0.016	0.032 ± 0.021	< 0.001	< 0.001	0.003
	Stance Time Variability	0.026 ± 0.015	0.034 ± 0.027	< 0.001	0.023	0.032
	Step Length Asymmetry	0.041 ± 0.019	0.050 ± 0.030	< 0.001	0.072	0.048
	Step Velocity Asymmetry	0.099 ± 0.062	0.106 ± 0.055	< 0.001	0.456	0.504
	Step Time Asymmetry	0.024 ± 0.015	0.036 ± 0.030	< 0.001	0.038	0.055
	Stride Time Asymmetry	0.015 ± 0.008	0.017 ± 0.021	< 0.001	0.438	0.363
	Swing Time Asymmetry	0.029 ± 0.028	0.036 ± 0.024	< 0.001	0.070	0.141
	Stance Time Asymmetry	0.029 ± 0.026	0.034 ± 0.022	< 0.001	0.146	0.247
	Step Length Coefficient of variation (CV)	0.065 ± 0.032	0.087 ± 0.042	< 0.001	0.001	0.001
	Step Velocity (CV)	0.076 ± 0.040	0.101 ± 0.052	< 0.001	0.001	0.002
	Step Time (CV)	0.045 ± 0.029	0.061 ± 0.045	< 0.001	0.006	0.011
	Stride Time (CV)	0.018 ± 0.007	0.027 ± 0.026	< 0.001	0.010	0.007
	Swing Time (CV)	0.057 ± 0.041	0.079 ± 0.052	< 0.001	0.001	0.007
	Stance Time (CV)	0.037 ± 0.025	0.047 ± 0.037	< 0.001	0.021	0.057
Signals power spectral density	Dominant Frequency (AP)	1.879 ± 0.385	1.851 ± 0.292	< 0.001	0.110	0.63
	Dominant Frequency (R)	1.886 ± 0.171	1.888 ± 0.330	< 0.001	0.124	0.973
	Dominant Frequency (ML)	4.269 ± 1.724	4.012 ± 2.283	0.001	0.354	0.463
	Dominant Frequency (V)	1.986 ± 0.575	1.930 ± 0.451	< 0.001	0.090	0.518
	Normalized Amplitude of dominant peak of Power Spectral Density (PSD) (AP)	0.035 ± 0.007	0.036 ± 0.007	0.126	0.319	0.444
	Normalized Amplitude of dominant peak of PSD (R)	0.044 ± 0.007	0.041 ± 0.009	0.001	0.019	0.010
	Normalized Amplitude of dominant peak of PSD (ML)	0.020 ± 0.008	0.017 ± 0.006	< 0.001	0.037	0.019
	Normalized Amplitude of dominant peak of PSD (V)	0.041 ± 0.009	0.038 ± 0.009	0.012	0.034	0.037
	Width of dominant peak of PSD (AP)	0.481 ± 0.005	0.482 ± 0.005	0.204	0.403	0.259
	Width of dominant peak of PSD (R)	0.482 ± 0.005	0.482 ± 0.010	< 0.001	0.561	0.766
	Width of dominant peak of PSD ML)	0.510 ± 0.038	0.512 ± 0.050	< 0.001	0.419	0.756
	Width of dominant peak of PSD (V)	0.483 ± 0.005	0.483 ± 0.012	< 0.001	0.662	0.883
	Slope of dominant peak of PSD (AP)	0.073 ± 0.015	0.075 ± 0.015	0.284	0.360	0.383
	Slope of dominant peak of PSD (R)	0.092 ± 0.015	0.085 ± 0.019	0.013	0.004	0.003
	Slope of dominant peak of PSD (ML)	0.041 ± 0.017	0.035 ± 0.013	< 0.001	0.021	0.011
	Slope of dominant peak of PSD (V)	0.085 ± 0.019	0.078 ± 0.020	0.064	0.010	0.012

Range of dominant peak of PSD (AP)	0.918 ± 0.223	0.780 ± 0.204	0.004	0.001	0.001
Range of dominant peak of PSD (R)	1.263 ± 0.348	1.120 ± 0.306	< 0.001	0.015	0.010
Range of dominant peak of PSD (ML)	0.991 ± 0.303	0.843 ± 0.264	< 0.001	0.005	0.003
Range of dominant peak of PSD (V)	1.191 ± 0.328	1.065 ± 0.296	< 0.001	0.030	0.018
PSD Amplitude (AP)	1.068 ± 0.215	1.102 ± 0.221	0.318	0.313	0.354
PSD Amplitude (R)	1.394 ± 0.226	1.255 ± 0.289	0.011	0.004	0.002
PSD Amplitude (ML)	0.608 ± 0.231	0.515 ± 0.170	< 0.001	0.016	0.006
PSD Amplitude (V)	1.281 ± 0.278	1.156 ± 0.292	0.078	0.010	0.011
PSD Mean Power (AP)	0.020 ± 0.001	0.021 ± 0.001	0.001	0.534	0.350
PSD Mean Power (R)	0.021 ± 0.001	0.021 ± 0.002	0.005	0.094	0.149
PSD Mean Power (ML)	0.020 ± 0.001	0.020 ± 0.002	0.013	0.213	0.262
PSD Mean Power (V)	0.021 ± 0.001	0.021 ± 0.001	0.006	0.045	0.072
PSD Median Power (AP)	0.001 ± 0.001	0.001 ± 0.001	0.001	0.534	0.350
PSD Median Power (R)	0.001 ± 0.001	0.001 ± 0.001	0.005	0.094	0.149
PSD Median Power (ML)	0.001 ± 0.001	0.001 ± 0.001	0.013	0.213	0.262
PSD Median Power (V)	0.001 ± 0.001	0.001 ± 0.001	0.006	0.045	0.072
PSD Normalized Width (AP)	0.481 ± 0.005	0.482 ± 0.005	0.204	0.403	0.259
PSD Normalized Width (R)	0.482 ± 0.005	0.482 ± 0.010	< 0.001	0.561	0.766
PSD Normalized Width (ML)	0.510 ± 0.038	0.512 ± 0.05	< 0.001	0.419	0.756
PSD Normalized Width (V)	0.483 ± 0.005	0.483 ± 0.012	< 0.001	0.662	0.883
PSD Normalized Slope (AP)	0.073 ± 0.015	0.075 ± 0.015	0.284	0.360	0.383
PSD Normalized Slope (R)	0.092 ± 0.015	0.085 ± 0.019	0.013	0.004	0.003
PSD Normalized Slope (ML)	0.041 ± 0.017	0.035 ± 0.013	< 0.001	0.021	0.011
PSD Normalized Slope (V)	0.085 ± 0.019	0.078 ± 0.020	0.064	0.010	0.012
PSD Normalized Range (AP)	0.918 ± 0.223	0.780 ± 0.204	0.004	0.001	0.001
PSD Normalized Range (R)	1.263 ± 0.348	1.120 ± 0.306	< 0.001	0.015	0.010
PSD Normalized Range (ML)	0.991 ± 0.330	0.843 ± 0.264	< 0.001	0.005	0.003
PSD Normalized Range (V)	1.191 ± 0.328	1.065 ± 0.296	< 0.001	0.030	0.018
PSD Integrated Power (AP)	30.610 ± 1.321	30.906 ± 2.177	0.001	0.534	0.350
PSD Integrated Power (R)	31.438 ± 1.559	30.944 ± 2.282	0.005	0.094	0.149
PSD Integrated Power (ML)	30.285 ± 1.767	29.851 ± 2.585	0.013	0.213	0.262
PSD Integrated Power (V)	31.458 ± 1.523	30.857 ± 2.224	0.006	0.045	0.072
Harmonic Ratio (AP)	3.502 ± 1.248	2.836 ± 0.949	0.146	0.001	0.001
Harmonic Ratio (R)	3.829 ± 1.173	3.287 ± 1.18	0.015	0.005	0.007
Harmonic Ratio (ML)	2.360 ± 0.862	1.994 ± 0.642	< 0.001	0.006	0.004
Harmonic Ratio (V)	3.760 ± 1.214	3.110 ± 1.091	0.017	0.001	0.001
Index of Harmonicity (AP)	0.630 ± 0.143	0.683 ± 0.136	< 0.001	0.010	0.028
Index of Harmonicity (R)	0.747 ± 0.103	0.678 ± 0.171	< 0.001	0.011	0.005
Index of Harmonicity (ML)	0.136 ± 0.166	0.243 ± 0.189	< 0.001	< 0.001	0.001
Index of Harmonicity (V)	0.719 ± 0.143	0.670 ± 0.177	< 0.001	0.061	0.076
Root Mean Square (RMS) of Straight Walk (AP)	0.163 ± 0.035	0.136 ± 0.036	0.003	< 0.001	< 0.001

Signal Magnitude	RMS Straight Walk (R)	0.325 ± 0.067	0.271 ± 0.076	0.001	< 0.001	< 0.001
	RMS Straight Walk (ML)	0.154 ± 0.049	0.123 ± 0.032	< 0.001	< 0.001	< 0.001
	RMS Straight Walk (V)	0.231 ± 0.052	0.197 ± 0.068	< 0.001	< 0.001	0.001
	RMS Stride (AP)	0.159 ± 0.035	0.133 ± 0.035	0.001	< 0.001	< 0.001
	RMS Stride (R)	0.302 ± 0.067	0.265 ± 0.075	0.001	< 0.001	< 0.001
	RMS Stride (ML)	0.151 ± 0.050	0.119 ± 0.032	< 0.001	0.001	< 0.001
	RMS Stride (V)	0.228 ± 0.052	0.193 ± 0.066	0.001	< 0.001	0.001
	RMS Step (AP)	0.143 ± 0.033	0.120 ± 0.033	0.001	< 0.001	0.001
	RMS Step (R)	0.293 ± 0.063	0.243 ± 0.070	0.001	< 0.001	< 0.001
	RMS Step (ML)	0.142 ± 0.049	0.111 ± 0.033	< 0.001	0.001	< 0.001
	RMS Step (V)	0.206 ± 0.046	0.175 ± 0.060	< 0.001	< 0.001	0.001
	RMS Ratio Straight Walk (AP)	0.504 ± 0.062	0.509 ± 0.070	0.595	0.319	0.713
	RMS Ratio Straight Walk (ML)	0.469 ± 0.088	0.460 ± 0.072	0.150	0.689	0.515
	RMS Ratio Straight Walk (V)	0.712 ± 0.076	0.717 ± 0.067	0.070	0.970	0.680
	RMS Ratio Stride (AP)	0.501 ± 0.063	0.507 ± 0.069	0.514	0.234	0.580
	RMS Ratio Stride (ML)	0.466 ± 0.092	0.457 ± 0.072	0.309	0.764	0.505
	RMS Ratio Stride (V)	0.714 ± 0.079	0.718 ± 0.067	0.062	0.866	0.721
	RMS Ratio Step (AP)	0.493 ± 0.068	0.502 ± 0.071	0.520	0.319	0.444
	RMS Ratio Step (ML)	0.479 ± 0.094	0.460 ± 0.074	0.571	0.317	0.183
	RMS Ratio Step (V)	0.701 ± 0.078	0.711 ± 0.067	0.158	0.604	0.401
Signal Regularity	Step Regularity (AP)	0.761 ± 0.191	0.712 ± 0.169	< 0.001	0.001	0.105
	Step Regularity (R)	0.595 ± 0.145	0.541 ± 0.148	< 0.001	0.012	0.029
	Step Regularity (ML)	0.541 ± 0.144	0.456 ± 0.135	0.087	0.001	0.001
	Step Regularity (V)	0.796 ± 0.143	0.741 ± 0.177	< 0.001	0.004	0.049
	Stride Regularity (AP)	0.835 ± 0.077	0.804 ± 0.105	< 0.001	0.049	0.055
	Stride Regularity (R)	0.688 ± 0.100	0.652 ± 0.110	0.001	0.032	0.045
	Stride Regularity (ML)	0.688 ± 0.121	0.616 ± 0.145	0.001	0.005	0.002
	Stride Regularity (V)	0.847 ± 0.115	0.813 ± 0.117	< 0.001	0.011	0.082
	Symmetry Autocorrelation Ratio (AP)	0.013 ± 0.029	0.016 ± 0.024	< 0.001	0.043	0.466
	Symmetry Autocorrelation Ratio (R)	0.016 ± 0.022	0.019 ± 0.023	< 0.001	0.139	0.380
	Symmetry Autocorrelation Ratio (ML)	0.174 ± 0.215	0.189 ± 0.382	< 0.001	0.247	0.777
	Symmetry Autocorrelation Ratio (V)	0.009 ± 0.011	0.012 ± 0.023	< 0.001	0.876	0.274
	Symmetry Autocorrelation Difference (AP)	0.099 ± 0.157	0.114 ± 0.132	< 0.001	0.036	0.547
	Symmetry Autocorrelation Difference (R)	0.121 ± 0.110	0.137 ± 0.110	< 0.001	0.149	0.411
	Symmetry Autocorrelation Difference (ML)	0.171 ± 0.105	0.189 ± 0.122	< 0.001	0.484	0.355
	Symmetry Autocorrelation Difference (V)	0.067 ± 0.058	0.095 ± 0.126	< 0.001	0.235	0.110
	Gait Symmetry Index	0.644 ± 0.083	0.637 ± 0.077	< 0.001	0.162	0.592
	Stride Harmonic Ratio (AP)	2.332 ± 0.552	2.049 ± 0.406	0.529	0.001	0.001
	Stride Harmonic Ratio (ML)	1.843 ± 0.510	1.584 ± 0.365	0.005	0.001	0.001
	Stride Harmonic Ratio (V)	2.280 ± 0.478	2.088 ± 0.416	0.107	0.003	0.011
	Jerk RMS of Straight walk (AP)	5.638 ± 1.900	4.802 ± 1.642	< 0.001	0.008	0.006

	Jerk RMS of Straight walk (R)	7.086 ± 2.622	6.198 ± 2.451	< 0.001	0.041	0.040
	Jerk RMS of Straight walk (ML)	6.354 ± 2.444	5.500 ± 1.907	< 0.001	0.025	0.021
	Jerk RMS of Straight walk (V)	7.615 ± 2.696	6.659 ± 2.690	< 0.001	0.016	0.038
	Jerk RMS of Stride (AP)	5.656 ± 1.911	4.808 ± 1.658	< 0.001	0.007	0.005
	Jerk RMS of Stride (R)	7.098 ± 2.613	6.178 ± 2.432	< 0.001	0.033	0.032
	Jerk RMS of Stride (ML)	6.341 ± 2.471	5.487 ± 1.896	< 0.001	0.030	0.021
	Jerk RMS of Stride (V)	7.638 ± 2.731	6.660 ± 2.678	< 0.001	0.014	0.034
	Jerk RMS of Step (AP)	5.571 ± 1.856	4.752 ± 1.610	< 0.001	0.044	0.041
	Jerk RMS of Step (R)	6.942 ± 2.486	6.095 ± 2.308	< 0.001	0.321	0.234
	Jerk RMS of Step (ML)	6.228 ± 2.42	5.391 ± 1.865	< 0.001	0.133	0.083
	Jerk RMS of Step (V)	7.510 ± 2.657	6.596 ± 2.643	< 0.001	0.194	0.155
	Jerk Mean Ratio of Straight Walk (AP)	-0.895 ± 4.075	0.215 ± 4.124	< 0.001	0.012	0.113
	Jerk Mean Ratio of Straight Walk (ML)	-0.287 ± 4.151	0.476 ± 2.885	< 0.001	0.207	0.199
	Jerk Mean Ratio of Straight Walk (V)	-0.421 ± 5.235	0.584 ± 2.912	< 0.001	0.396	0.148
	Jerk Mean Ratio of Stride (AP)	0.361 ± 4.626	1.477 ± 11.974	< 0.001	0.951	0.491
	Jerk Mean Ratio of Stride (ML)	-0.507 ± 3.311	-0.532 ± 4.589	< 0.001	0.550	0.971
	Jerk Mean Ratio of Stride (V)	1.265 ± 5.900	2.498 ± 17.561	< 0.001	0.561	0.600
	Jerk Mean Ratio of Step (AP)	0.442 ± 5.292	-2.729± 27.314	< 0.001	0.846	0.373
	Jerk Mean Ratio of Step (ML)	-2.303 ± 26.784	-20.711± 190.467	< 0.001	0.531	0.455
Signal Complexity	Jerk Mean Ratio of Step (V)	-0.702 ± 9.174	-7.326 ± 61.82	< 0.001	0.443	0.408
	Jerk Mean Log Ratio of Straight Walk (AP)	-1.305 ± 0.959	-1.344 ± 1.026	0.562	0.961	0.818
	Jerk Mean Log Ratio of Straight Walk (ML)	-0.83 ± 1.021	-0.759 ± 1.212	0.402	0.872	0.712
	Jerk Mean Log Ratio of Stride (AP)	1073.052 ± 8391.063	-1.344 ± 1.015	< 0.001	0.869	0.251
	Jerk Mean Log Ratio of Stride (ML)	1073.497 ± 8391.007	-0.785 ± 1.208	< 0.001	0.977	0.251
	Jerk Mean Log Ratio of Step (AP)	1073.065 ± 8391.063	-1.367 ± 0.997	< 0.001	0.683	0.251
	Jerk Mean Log Ratio of Step (ML)	1073.488 ± 8391.008	-0.841 ± 1.205	< 0.001	0.817	0.251
	Jerk Max of Straight Walk (AP)	18.144 ± 7.931	16.159 ± 6.020	< 0.001	0.209	0.092
	Jerk Max of Straight Walk (R)	27.934 ± 12.564	25.984± 11.126	< 0.001	0.426	0.330
	Jerk Max of Straight Walk (ML)	23.190 ± 9.934	21.202 ± 7.419	< 0.001	0.347	0.174
	Jerk Max of Straight Walk (V)	29.193 ± 12.821	26.007± 11.094	< 0.001	0.178	0.116
	Jerk Max of Stride (AP)	15.111 ± 6.843	13.057 ± 5.251	< 0.001	0.074	0.045
	Jerk Max of Stride (R)	23.495 ± 10.674	21.456 ± 9.727	< 0.001	0.252	0.238
	Jerk Max of Stride (ML)	19.227 ± 8.738	17.197 ± 6.325	< 0.001	0.223	0.110
	Jerk Max of Stride(V)	24.484 ± 11.627	21.449 ± 9.446	< 0.001	0.135	0.088
	Jerk Max of Step (AP)	12.622 ± 5.227	10.849 ± 4.157	< 0.001	0.044	0.026
	Jerk Max of Step (R)	20.046 ± 9.319	18.283 ± 7.951	< 0.001	0.315	0.227
	Jerk Max of Step (ML)	16.295 ± 7.623	14.373 ± 5.302	< 0.001	0.141	0.079
	Jerk Max of Step (V)	20.481 ± 9.318	18.457 ± 8.286	< 0.001	0.199	0.174

Jerk Min of Straight Walk (AP)	-23.532 ± 9.171	-21.972± 7.619	< 0.001	0.422	0.270
Jerk Min of Straight Walk (R)	-27.921 ± 11.039	-25.687 ± 10.183	< 0.001	0.219	0.214
Jerk Min of Straight Walk (ML)	-23.271 ± 9.447	-21.665± 8.745	< 0.001	0.264	0.297
Jerk Min of Straight Walk (V)	-27.322 ± 11.62	-25.183 ± 10.283	< 0.001	0.398	0.248
Jerk Min of Stride (AP)	-20.382 ± 8.191	-18.6 ± 6.762	< 0.001	0.247	0.158
Jerk Min of Stride (R)	-23.145 ± 9.566	-21.143± 8.835	< 0.001	0.277	0.199
Jerk Min of Stride (ML)	-19.309 ± 8.436	-17.352± 7.036	< 0.001	0.138	0.134
Jerk Min of Stride (V)	-23.15 ± 10.192	-21.009± 8.893	< 0.001	0.271	0.185
Jerk Min of Step (AP)	-17.956 ± 7.244	-16.092± 5.781	< 0.001	0.182	0.090
Jerk Min of Step (R)	-19.273 ± 7.817	-17.833± 7.268	< 0.001	0.350	0.260
Jerk Min of Step (ML)	-16.095 ± 7.041	-14.326± 5.491	< 0.001	0.118	0.095
Jerk Min of Step (V)	-19.874 ± 8.604	-17.936± 7.704	< 0.001	0.203	0.161
Jerk Range of Straight Walk (AP)	41.676 ± 15.642	38.131± 13.121	< 0.001	0.173	0.145
Jerk Range of Straight Walk (R)	55.855 ± 23.125	51.671± 20.627	< 0.001	0.327	0.258
Jerk Range of Straight Walk (ML)	46.460 ± 18.960	42.868± 15.643	< 0.001	0.242	0.218
Jerk Range of Straight Walk (V)	56.515 ± 23.868	51.190± 20.322	< 0.001	0.229	0.154
Jerk Range of Stride (AP)	35.493 ± 13.622	31.656± 11.588	< 0.001	0.068	0.072
Jerk Range of Stride (R)	46.64 ± 19.846	42.599± 18.067	< 0.001	0.252	0.208
Jerk Range of Stride (ML)	38.536 ± 16.848	34.549± 13.027	< 0.001	0.139	0.114
Jerk Range of Stride (V)	47.634 ± 21.333	42.459± 17.514	< 0.001	0.206	0.115
Jerk Range of Step (AP)	30.578 ± 11.385	26.941 ± 9.6	< 0.001	0.044	0.041
Jerk Range of Step (R)	39.318 ± 16.896	36.116 ± 14.94	< 0.001	0.321	0.234
Jerk Range of Step (ML)	32.389 ± 14.503	28.699± 10.655	< 0.001	0.133	0.083
Jerk Range of Step (V)	40.355 ± 17.546	36.393± 15.372	< 0.001	0.194	0.155
Lyapunov RC (AP)	1150.261 ± 8385.92	874.373 ± 7277.96	< 0.001	0.150	0.834
Lyapunov RC (R)	0.619 ± 0.138	0.642 ± 0.159	0.050	0.456	0.369
Lyapunov RC (ML)	0.574 ± 0.117	0.573 ± 0.139	0.006	0.773	0.960
Lyapunov RC (V)	0.608 ± 0.142	862.921 ± 7278.602	< 0.001	0.212	0.357
Lyapunov W (AP)	1.284 ± 0.265	1.436 ± 0.444	0.001	0.028	0.019
Lyapunov W (R)	1.083 ± 0.312	1.299 ± 0.465	0.001	0.007	0.002
Lyapunov W (ML)	1.806 ± 0.473	2.001 ± 0.384	0.035	0.135	0.053
Lyapunov W (V)	1.151 ± 0.377	1.330 ± 0.452	0.002	0.020	0.013
Full Orbit Eccentricity	0.991 ± 0.006	0.981 ± 0.027	< 0.001	0.001	0.005
Relative Orbit Inclination	6.315 ± 3.436	7.371 ± 4.822	< 0.001	0.451	0.148
Full Orbit Area	0.138 ± 0.105	0.126 ± 0.12	< 0.001	0.022	0.534
Full Orbit Minor axis SD	0.023 ± 0.016	0.027 ± 0.023	< 0.001	0.739	0.274
Full Orbit Major axis SD	0.089 ± 0.123	0.082 ± 0.101	< 0.001	0.553	0.702
Short Half Orbit Eccentricity Asymmetry	0.015 ± 0.018	0.026 ± 0.034	< 0.001	0.024	0.018

Short Half Orbit Segment angle	4.942 ± 2.713	5.985 ± 3.99	< 0.001	0.025	0.006
Short Half Orbit Area Asymmetry	25.906 ± 136.824	47.472 ± 248.117	< 0.001	0.209	0.541
Long Half Orbit Eccentricity Asymmetry	0.016 ± 0.016	0.023 ± 0.021	< 0.001	0.024	0.038
Long Half Orbit Segment Angle	4.942 ± 2.713	5.985 ± 3.99	< 0.001	0.106	0.081
Long Half Orbit Area Asymmetry	160.622 ± 877.581	16.141± 30.027	< 0.001	0.323	0.141
Intra-Step Correlation	0.968 ± 0.022	0.934 ± 0.084	< 0.001	< 0.001	0.003

TABLE S3.
DEFINITIONS OF THE GAIT CHARACTERISTICS USED IN THE STUDY

Domain	Feature	Definition
Spatial-temporal[5]	Step Length	Distance between two heel strikes
	Step Velocity	Step length/step time

	Step & Stride Time	Time between two heel strikes & two consecutive heel strikes of same foot
	Swing Time	Time between toe-off to heel strike
	Stance Time	Time between heel strike to toe-off
	Cadence	Number of steps per minute
	Variability (SD & CV)<br>- Step Length<br>- Step Velocity<br>- Step & Stride Time<br>- Swing & Stance Time	Standard deviation (SD) and coefficient of variation (CV:SD/mean) of the extracted mean spatial-temporal gait characteristics
	Asymmetry<br>- Step Length<br>- Step Velocity<br>- Step & Stride Time<br>- Swing & Stance Time	Difference between the left and right feet of the extracted mean spatial-temporal gait characteristics
Signals Power Spectral Density	Dominant Frequency	The inverse of the period at which the maximum spectral response is observed. [6, 7]
	Normalized Amplitude of dominant peak of Power Spectral Density (PSD) in the three axes (V, AP, ML, & R)	Estimated from each detrended acceleration signal (VT, ML, AP) as the magnitude of the PSD at the dominant peak, normalized by the total integrated PSD. This feature represents the relative strength of the signal at the most dominant frequency and reflects the periodicity of the signal. Larger values of this feature indicate a greater periodic gait pattern. [6, 7]
	Width of dominant peak of PSD (V, AP, ML, & R)	Estimated from each detrended acceleration signal (VT, ML, AP) as the width of the dominant peak of the PSD. This feature is a measure of frequency dispersion and is related to the variability of the dominant cycles of the signal (step cycles in VT and AP, stride cycles in ML). Larger values of this feature indicate a less consistent gait pattern. [7]
	- Slope of dominant peak of PSD (V, AP, ML, & R)	Estimated from each detrended acceleration signal (VT, ML, AP) as the slope of the dominant peak of the PSD. [6]
	- Range of dominant peak of PSD (V, AP, ML, & R)	Estimated from each detrended acceleration signal (VT, ML, AP) as the range of the dominant peak of the PSD. [6]
	- PSD Mean Power (V, AP, ML, & R)	Estimated from each detrended acceleration signal (VT, ML & AP) as the mean power of the PSD. [8]
	- PSD Median Power (V, AP, ML, & R)	Estimated from each detrended acceleration signal (VT, ML & AP) as the median power of the PSD. [8]
	- PSD Normalized Width (V, AP, ML, & R)	Estimated from each detrended acceleration signal (VT, ML, AP) as the width of the dominant peak of the PSD, normalized by the total integrated PSD.
	- PSD Normalized Slope (V, AP, ML, & R)	Estimated from each detrended acceleration signal (VT, ML & AP) as the slope of the dominant peak of the PSD, normalized by the total integrated PSD.
	- PSD Normalized Range (V, AP, ML, & R)	Estimated from each detrended acceleration signal (VT, ML & AP) as the range of the dominant peak of the PSD, normalized by the total integrated PSD.
	- PSD Integrated Power (V, AP, ML, & R)	Estimated from each detrended acceleration signal (VT, ML & AP) as the total integration of the PSD. [9]
	- Harmonic Ratio Straight walk (V, AP, ML, & R)	Estimated for each direction as described by Menz et al., 2003 [10, 11]. Harmonic ratios of acceleration signals in VT and AP directions were calculated as the sum of even harmonics divided by the sum of odd harmonics, since these signals have two phases per stride. Harmonic ratios from ML acceleration were calculated as the sum of odd harmonics divided by the sum of even harmonics, since acceleration signals in mediolateral (ML) direction are monophasic per stride. This measurement reflects the rhythmicity of periodic patterns and relates to gait symmetry [12]. Thus, higher values of this feature are related to more rhythmic, paced and symmetric gait patterns [13].
	Harmonic Ratio<br>- Stride Harmonic Ratio (V, ML, & AP)	The step-to-step symmetry within a stride from calculating a ratio of the odd and even harmonics of a signal following fast Fourier transformation [10].

	- Index of Harmonicity (V, AP, ML, & R)	Estimated for each direction as the PSD of the fundamental frequency (first harmonic) divided by the cumulative sum of the power spectral density of the first six harmonics [13, 14]. This measure, proposed by Lamoth et al., 2002 reflects gait smoothness [13]. Thus, values approaching the maximum value of 1.0 indicate a smoother gait pattern, which may reflect a less vigorous/more cautious movement pattern, whereas smaller values might indicate movements that are more erratic.
Signal Magnitude	Root Mean Square (RMS)<br>- RMS of Straight Walk (V, AP, ML, & R)<br>- RMS Stride (V, AP, ML, & R)<br>- RMS Step (V, AP, ML, & R)<br>- RMS Ratio Straight Walk (V, AP, & ML)<br>- RMS Ratio Stride (V, AP, & ML)<br>- RMS Ratio Step (V, AP, & ML)	Acceleration RMS:<br><br>The calculation of the Root mean square of the acceleration signal [15].
	Jerk<br>- RMS of Straight Walk (V, AP, ML, & R)<br>- RMS of Stride (V, AP, ML, & R)<br>- RMS of Step (V, AP, ML, & R)	Jerk RMS:<br><br>The calculation of the root mean square of the first time derivative of the acceleration signal (jerk) [16].
	- Mean Ratio of Straight Walk (V, AP, & ML)<br>- Mean Ratio of Stride (V, AP, & ML)<br>- Mean Ratio of Step (V, AP, & ML)<br>- Mean Log Ratio of Straight Walk (AP, & ML)<br>- Mean Log Ratio of Stride (AP, & ML)<br>- Mean Log Ratio of Step (AP, & ML)<br>- Max of Straight Walk (V, AP, ML, & R)<br>- Max of Stride (V, AP, ML, & R)<br>- Max of Step (V, AP, ML, & R)<br>- Min of Straight Walk (V, AP, ML, & R)<br>- Min of Stride (V, AP, ML, & R)<br>- Min of Step (V, AP, ML, & R)<br>- Range of Straight Walk (V, AP, ML, & R)<br>- Range of Stride (V, AP, ML, & R)<br>- Range of Step (V, AP, ML, & R)	Jerk ratio:<br><br>A logarithmic ratio of either the AP or ML acceleration RMS over the V acceleration RMS [16, 17]
Signal Regularity	Step Regularity (V, AP, ML & R)	Estimated as the normalized unbiased auto-covariance for a lag of one step time [17]. This feature thus reflects the similarity between subsequent steps of the acceleration pattern over a step. Values of this feature close to 1.0 (maximum possible value) reflect repeatable patterns between subsequent steps.
	Stride Regularity (V, AP, ML, & R)	Estimated as the normalized unbiased auto-covariance for a lag of one stride time [17]. This feature thus reflects the similarity between subsequent strides of the acceleration pattern over a stride cycle.
	Symmetry Autocorrelation Ratio (V, AP, ML, & R)	A ratio between step and stride regularity designed to quantify the level of symmetry between them and indicative of symmetry during a straight walk [17].
	Symmetry Autocorrelation Difference (V, AP, ML, & R)	Difference between step and stride regularity designed to quantify the level of symmetry between them and indicative of symmetry during a straight walk [17].
	Gait Symmetry Index	Calculated based upon the concept of the summation of the biased autocorrelation from all three components of movement and a subsequent calculation of step and stride timing asymmetry [18].

	Lyapunov exponent	Estimated as the exponential rate of divergence or convergence after a small disturbance of nearby orbits in state space. Since nearby orbits correspond to nearly identical states, a positive value indicates that systems with initial differences will soon behave quite differently, and stability is low [19].<br>The Lyapunov exponent was calculated using the Rostein and Wolf method [20] for each detrended acceleration signal (VT, ML & AP). We used an embedding dimension of 5 and a delay of 12 samples [21].<br>As local dynamic stability estimates based on a 6D and 12D state space correlated highly with those of the employed 9D state space in a previous study [22] (respectively r≥0.94 and r≥0.81), the number of embedding dimensions has been considered to have minor effects [22]. Thus, to reduce computational cost, we explored the use of a 5D state reconstruction (with two less dimensions than used in the reference) [23].
	Phase plots features [24]:	
	Features from ellipses fitted to full cycles/orbits of phase plots:	
	Full orbit eccentricity	Average eccentricity of all fully fitted ellipses.
	Relative orbit inclination	Average angle subtended by alternating fitted ellipses within a bout of gait.
	Full orbit area	Average area of all fully fitted ellipses<br>(A = π * minor axis * major axis).
	Full orbit minor axis SD	SD of minor axes lengths of all fully fitted ellipses. Analogous to PCA (Second component)
	Full orbit major axis SD	SD of major axes lengths of all fully fitted ellipses. Analogous to PCA (First component).
Signal Complexity	Features from ellipses fitted to half-cycles/orbits following ellipse segmentation along the minor (short) axis:	
	Short half orbit eccentricity asymmetry	Difference in eccentricity of two ellipses fitted to each half-cycle of a full orbit in the phase plot. Averaged over all orbits in bout's phase plot.
	Short half orbit segment angle	Difference in inclination of two ellipses fitted to each half-cycle of a full orbit in the phase plot. Averaged over all orbits in bout's phase plot.
	Short half orbit area asymmetry	Difference in area of two ellipses fitted to each half-cycle of a full orbit in the phase plot. Averaged over all orbits in bout's phase plot.
	Features from ellipses fitted to half-cycles/orbits following ellipse segmentation along the major (long) axis:	
	Long half orbit eccentricity asymmetry	Difference in eccentricity of two ellipses fitted to each half-cycle of a full orbit in the phase plot. Averaged over all orbits in bout's phase plot.
	Long half orbit segment angle	Difference in inclination of two ellipses fitted to each half-cycle of a full orbit in the phase plot. Averaged over all orbits in bout's phase plot.
	Long half orbit area asymmetry	Difference in area of two ellipses fitted to each half-cycle of a full orbit in the phase plot. Averaged over all orbits in bout's phase plot.
	Intra-step correlation	Average correlation of acceleration signal corresponding to step i with that of step i-1. I.e. a lag-1 autocorrelation where a single lag is one step cycle's duration.

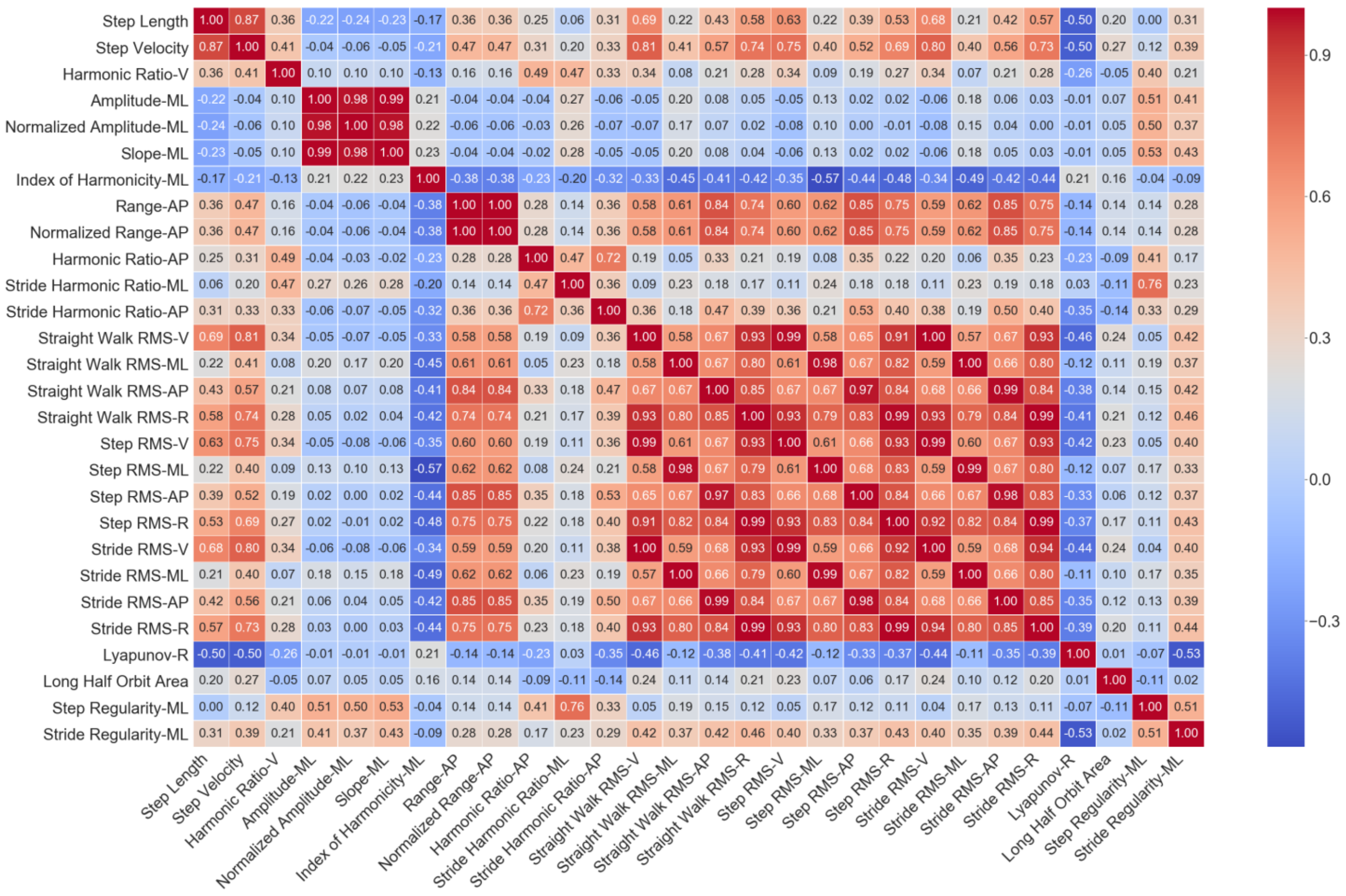

Fig. S2. Correlation among important characteristics having variable importance score above 1.5

References

[1] C. Caramia *et al.*, "IMU-Based Classification of Parkinson's Disease From Gait: A Sensitivity Analysis on Sensor Location and Feature Selection," *IEEE journal of biomedical and health informatics,* vol. 22, no. 6, pp. 1765-1774, 2018.

[2] S. Maitra and J. Yan, "Principle component analysis and partial least squares: Two dimension reduction techniques for regression," *Applying Multivariate Statistical Models,* vol. 79, pp. 79-90, 2008.

[3] M. Fordellone, A. Bellincontro, and F. Mencarelli, "Partial least squares discriminant analysis: A dimensionality reduction method to classify hyperspectral data," *arXiv preprint arXiv:1806.09347,* 2018.

[4] L. Eriksson, T. Byrne, E. Johansson, J. Trygg, and C. Vikström, *Multi-and megavariate data analysis basic principles and applications*. Umetrics Academy, 2013.

[5] S. Del Din, A. Godfrey, and L. Rochester, "Validation of an accelerometer to quantify a comprehensive battery of gait characteristics in healthy older adults and Parkinson's disease: toward clinical and at home use," *IEEE journal of biomedical and health informatics,* vol. 20, no. 3, pp. 838-847, 2015.

[6] A. Weiss, S. Sharifi, M. Plotnik, J. P. van Vugt, N. Giladi, and J. M. Hausdorff, "Toward automated, at-home assessment of mobility among patients with Parkinson disease, using a body-worn accelerometer," *Neurorehabilitation and neural repair,* vol. 25, no. 9, pp. 810-818, 2011.

[7] E. Micó-Amigo *et al.*, "Potential markers of progression in idiopathic Parkinson's disease derived from assessment of circular gait with a single body-fixed-sensor: A 5-year longitudinal study," *Frontiers in human neuroscience,* vol. 13, p. 59, 2019.

[8] Q. W. Oung, M. Hariharan, H. L. Lee, S. N. Basah, M. Sarillee, and C. H. Lee, "Wearable multimodal sensors for evaluation of patients with Parkinson disease," in *2015 IEEE International Conference on Control System, Computing and Engineering (ICCSCE)*, 2015, pp. 269-274: IEEE.

[9] A. Mannini, M. Rosenberger, W. L. Haskell, A. M. Sabatini, and S. S. Intille, "Activity recognition in youth using single accelerometer placed at wrist or ankle," *Medicine and science in sports and exercise,* vol. 49, no. 4, p. 801, 2017.

[10] J. Bellanca, K. Lowry, J. VanSwearingen, J. Brach, and M. Redfern, "Harmonic ratios: a quantification of step to step symmetry," *Journal of biomechanics,* vol. 46, no. 4, pp. 828-831, 2013.

[11] H. B. Menz, S. R. Lord, and R. C. Fitzpatrick, "Acceleration patterns of the head and pelvis when walking on level and irregular surfaces," *Gait & posture,* vol. 18, no. 1, pp. 35-46, 2003.

[12] I. Pasciuto, E. Bergamini, M. Iosa, G. Vannozzi, and A. Cappozzo, "Overcoming the limitations of the Harmonic Ratio for the reliable assessment of gait symmetry," *Journal of biomechanics,* vol. 53, pp. 84-89, 2017.

[13] C. J. Lamoth, O. G. Meijer, P. I. Wuisman, J. H. van Dieën, M. F. Levin, and P. J. Beek, "Pelvis-thorax coordination in the transverse plane during walking in persons with nonspecific low back pain," *Spine,* vol. 27, no. 4, pp. E92-E99, 2002.

[14] S. M. Rispens, M. Pijnappels, K. S. van Schooten, P. J. Beek, A. Daffertshofer, and J. H. van Dieën, "Consistency of gait characteristics as determined from acceleration data collected at different trunk locations," *Gait & posture,* vol. 40, no. 1, pp. 187-192, 2014.

[15] J. J. Kavanagh and H. B. Menz, "Accelerometry: a technique for quantifying movement patterns during walking," *Gait & posture,* vol. 28, no. 1, pp. 1-15, 2008.

[16] M. A. Brodie, H. B. Menz, and S. R. Lord, "Age-associated changes in head jerk while walking reveal altered dynamic stability in older people," *Experimental brain research,* vol. 232, no. 1, pp. 51-60, 2014.

[17] R. Moe-Nilssen and J. L. Helbostad, "Estimation of gait cycle characteristics by trunk accelerometry," *Journal of biomechanics,* vol. 37, no. 1, pp. 121-126, 2004.

[18] W. Zhang, M. Smuck, C. Legault, M. A. Ith, A. Muaremi, and K. Aminian, "Gait symmetry assessment with a low back 3d accelerometer in post-stroke patients," *Sensors,* vol. 18, no. 10, p. 3322, 2018.

[19] K. S. van Schooten *et al.*, "Sensitivity of trunk variability and stability measures to balance impairments induced by galvanic vestibular stimulation during gait," *Gait & posture,* vol. 33, no. 4, pp. 656-660, 2011.

[20] A. Dupeyron, S. M. Rispens, C. Demattei, and J. H. van Dieën, "Precision of estimates of local stability of repetitive trunk movements," *European Spine Journal,* vol. 22, no. 12, pp. 2678-2685, 2013.

[21] S. M. Rispens, K. S. van Schooten, M. Pijnappels, A. Daffertshofer, P. J. Beek, and J. H. van Dieen, "Identification of fall risk predictors in daily life measurements: gait characteristics' reliability and association with self-reported fall history," *Neurorehabilitation and neural repair,* vol. 29, no. 1, pp. 54-61, 2015.

[22] K. S. van Schooten, S. M. Rispens, P. J. Elders, J. H. van Dieën, and M. Pijnappels, "Toward ambulatory balance assessment: estimating variability and stability from short bouts of gait," *Gait & posture,* vol. 39, no. 2, pp. 695-699, 2014.

[23] P. Terrier and F. Reynard, "Maximum Lyapunov exponent revisited: Long-term attractor divergence of gait dynamics is highly sensitive to the noise structure of stride intervals," *Gait & posture,* vol. 66, pp. 236-241, 2018.

[24] M. Dunne-Willows, P. Watson, J. Shi, L. Rochester, and S. Del Din, "A Novel Parameterisation of Phase Plots for Monitoring of Parkinson's Disease," in *2019 41st Annual International Conference of the IEEE Engineering in Medicine and Biology Society (EMBC)*, 2019, pp. 5890-5893: IEEE.
